# Comprehensive Survey on Nanobiomaterials for Bone Tissue Engineering Applications

**DOI:** 10.3390/nano10102019

**Published:** 2020-10-13

**Authors:** Pawan Kumar, Meenu Saini, Brijnandan S. Dehiya, Anil Sindhu, Vinod Kumar, Ravinder Kumar, Luciano Lamberti, Catalin I. Pruncu, Rajesh Thakur

**Affiliations:** 1 Department of Materials Science and Nanotechnology, Deenbandhu Chhotu Ram University of Science and Technology, Murthal 131039, India; meenu.rschmsn@dcrustm.org (M.S.); drbrijdehiya.msn@dcrustm.org (B.S.D.); 2 Department of Biotechnology, Deenbandhu Chhotu Ram University of Science and Technology, Murthal 131039, India; sindhu.biotech@gmail.com; 3 Department of Bio and Nanotechnology, Guru Jambheshwar University of Science and Technology, Hisar 125001, India; indoravinod2@gmail.com (V.K.); rtnano@gmail.com (R.T.); 4 School of Mechanical Engineering, Lovely Professional University, Phagwara 144411, India; 5 Dipartimento di Meccanica, Matematica e Management, Politecnico di Bari, 70125 Bari, Italy; luciano.lamberti@poliba.it; 6 Department of Design, Manufacturing & Engineering Management, University of Strathclyde, Glasgow G1 1XJ, UK; 7 Department of Mechanical Engineering, Imperial College London, London SW7 2AZ, UK

**Keywords:** nano-biomaterials, nanotechnology, scaffolds, hard tissue engineering

## Abstract

One of the most important ideas ever produced by the application of materials science to the medical field is the notion of biomaterials. The nanostructured biomaterials play a crucial role in the development of new treatment strategies including not only the replacement of tissues and organs, but also repair and regeneration. They are designed to interact with damaged or injured tissues to induce regeneration, or as a forest for the production of laboratory tissues, so they must be micro-environmentally sensitive. The existing materials have many limitations, including impaired cell attachment, proliferation, and toxicity. Nanotechnology may open new avenues to bone tissue engineering by forming new assemblies similar in size and shape to the existing hierarchical bone structure. Organic and inorganic nanobiomaterials are increasingly used for bone tissue engineering applications because they may allow to overcome some of the current restrictions entailed by bone regeneration methods. This review covers the applications of different organic and inorganic nanobiomaterials in the field of hard tissue engineering.

## 1. Introduction

Nanobiomaterials denote nanometer-sized materials whose structures and constituents have significant and novel characteristics with a strong impact on healing and medicine [1,2]. They include metals, ceramics, polymers, hydrogels, and novel self-assembled materials [3]. Rapid developments in nanotechnology not only led to create new materials and tools for biomedical applications, but also changed the way of using these materials in science and technology [4,5]. 

**Figure 1:**
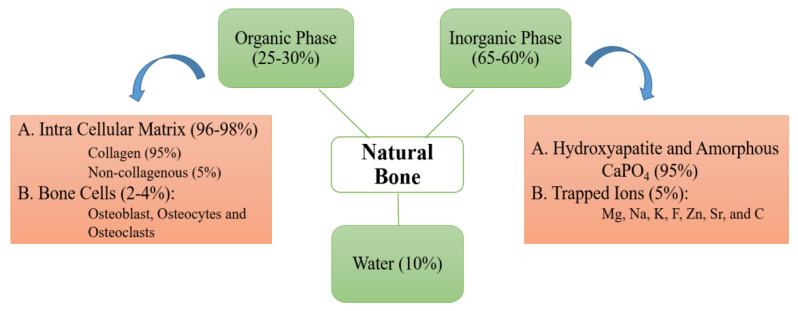
Composition of natural bone.

Human bone is a dynamic tissue that can rebuild and remodel in the body throughout life [6]. The human bone is a hierarchical assembly of nano- to macro-scale organic and inorganic components involved in transmitting physio-chemical and mechano-chemical cues [7,8]. The schematic of Figure 1 shows that normal human bone contains 30% organic collagen fibrils and 70% inorganic minerals [9,10,11,12], while 2% of the total volume is occupied by bone cells, osteoblasts, osteoclasts, lining cells, progenitor cells, and adipocytes [13,14]. Crystalline phases form 65% of the dry weight of the mineral matrix and most part of calcined fraction in calcium phosphate [15,16].

The continuously growing population and the higher complexity of human interactions have generated new bone-related diseases (e.g., bone tumors, bone infections, and bone loss). This requires effective handling and treatment for bone regeneration [17,18]. Tissue engineering has revolutionized orthopedic and surgical studies, providing a new direction in the field based on nanoscale surface modification to simulate properties of extracellular matrix (ECM) and new foundations of structural variables of autologous tissue [19,20]. Tissue engineering is used to generate, restore, and/or replace tissues and organs by using biomaterials and helps to produce similar native tissue or organ [21]. 

**Figure 2:**
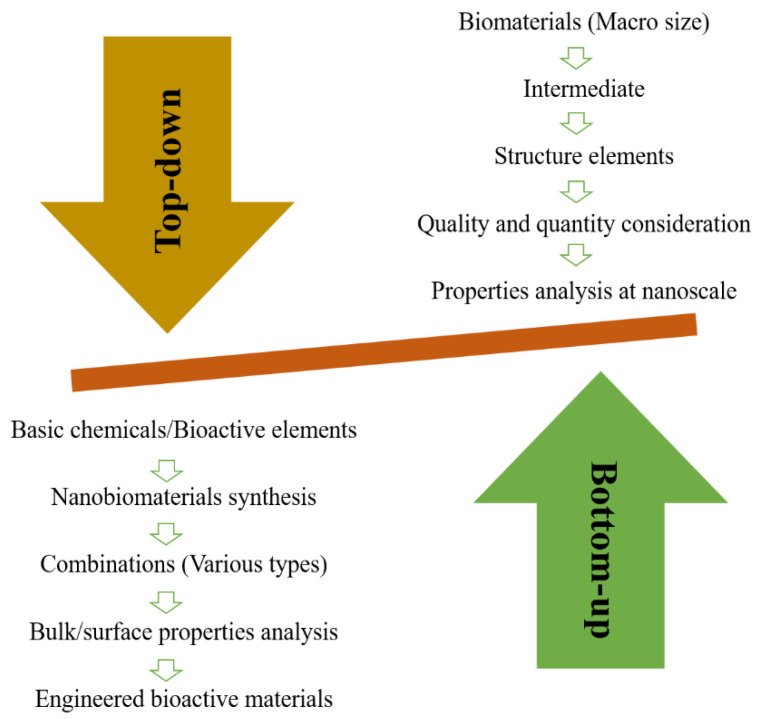
Design of bioactive materials based on top-down and bottom-up approaches.

Nanotechnology solved many questions in tissue engineering by modifying regenerative strategies [22]. Biomedical applications of nanotechnology became a hot subject because different nanomaterials are utilized for the synthesis of scaffolds or implants [23,24]. These nanomaterials may be metallic [25], ceramic, or polymeric [26] with different structural forms such as tubes, rods, fibers, and spheres [27]. Various properties of materials such as physiochemical, electrical, mechanical, optical, catalytic, and magnetic properties can be improved at the nanoscale [28] and tailored to specific applications. Nanomaterials synthesized through top-down or bottom-up approaches [29] (Figure 2) have outstanding properties, which are used for biomedical applications particularly in tissue engineering [22]. 

Existing biomaterials often do not integrate with host tissue completely. This may cause infection and foreign body reactions that lead to implant failure [30]. Indeed, nanostructured biomaterials imitate the natural bone’s extracellular matrix (ECM), producing an artificial microenvironment that promotes cell adhesion, proliferation and differentiation [31]. The specific biological, morphological, and biochemical properties of nanobiomaterials attract researchers to use them for the hard tissue engineering [32]. Nanostructured biomaterials can be used to fabricate high-performance scaffolds or implants with tailored physical, chemical, and biological properties. Several natural and synthetic nanostructured biomaterials are now available for the fabrication of scaffolds with decent bioactivity [33]. 

This survey article presents the most relevant applications of nanobiomaterials to bone tissue engineering, trying to highlight how organic and inorganic nanobiomaterials can deal with the above mentioned requirements on bone regeneration and the multiple challenges entailed by such a complicated subject. A broad overview of the various types of nanobiomaterials and their applications in the field of hard tissue engineering is provided.

Besides the introductory articles mentioned above and other general articles on topics related to nanomaterials and the different contexts where they operate, the present survey covers some 550 technical papers focusing on types, fabrication, and applications of nanobiomaterials. These articles have been selected using three widely used academic search engines: Scopus, Web of Science, and Google Scholar. For that purpose, keywords such as “tissue engineering”, “bone tissue engineering”, “tissue regeneration”, “scaffolds”, “nanomaterials”, and “nanobiomaterials” as well as their combinations have been used as input for the search process. High priority is given to peer-reviewed journal articles with respect to book chapters and conference proceedings, which count for some 10 papers, less than 1.75% of the total number of surveyed articles. More detailed statistics on the articles surveyed for each topic will be reported at the end of the corresponding subsections.

The paper is structured as follows. Section 2 and Section 3 describe the various types of organic and inorganic nanobiomaterials and their applications in bone tissue engineering/regenerative medicines, drug/gene delivery, anti-infection properties, coatings, scaffold fabrication, and cancer therapy; generalizing conclusions are given at the end of each section. The conclusion section summarizes the main findings of this survey.

## 2. Nanobiomaterials

Nanobiomaterials cover a wide variety of biomaterials including natural and artificial materials, used for various applications in tissue engineering [34,35,36]. These materials can be classified into two categories, i.e., organic nanobiomaterials and inorganic nanobiomaterials, where the former is characterized by the presence of carbon-containing constituents. Organic–inorganic hybrids are much more effective biomaterials than pure polymers, bioglasses, metals, alloys, and ceramics [37] as they try to combine best properties of constituents following the general concept of composite material. Section 2.1 will review the different types of organic nanobiomaterials, while Section 2.2 will review the different types of inorganic nanobiomaterials.

### 2.1. Organic Nanobiomaterials

Nanostructured materials have characteristics like biocompatibility, nontoxicity, and non-carcinogenicity. When used for replacement or restoration of body tissue, they are regarded as organic nanobiomaterials [38]. Several research groups have shifted their attention from metallic to organic nanomaterials, such as lipids, liposomes, dendrimers, and polymers including chitosan, gelatin, collagen, or other biodegradable polymers [39]. Organic materials are combinations of a few of the lightest elements, particularly hydrogen, nitrogen and oxygen, and carbon-containing chemical compounds located within living organisms [40]. Proteins, nucleic acids, lipids, and carbohydrates (the polysaccharides) are the basic types of organic materials [41].

Table 1 presents a general classification of organic nanobiomaterials and summarizes representative applications of each material in tissue engineering. The following subsections present a general description of each nanomaterial type listed in Table 1 and a detailed literature survey on the corresponding developments for tissue engineering.

#### 2.1.1. Lipids

Lipids are small hydrophobic or amphiphilic molecules [67]. They can be classified as fatty lipids of acylglycerol, phospholipids such as glycerides, seduction lipids, sterols, demonstrations of lipids played, lipids, and polylactide Kane [68]. Lipids are essential agents for the physiological and pathophysiological functioning of cells [69]. Generally, 10–1000 nm sized spherical lipid nanoparticles are synthesized [70]. All organisms consist of lipids as basic components, among other ingredients. The use of these lipids in pharmaceutical and biomedical fields can solve the problem of biocompatibility and biodegradation [71]. Besides liposomes (lipids arranged in the formation), other unique structures (e.g., hexagonal, spongy, solid structure, etc.) resulting from lipid polymorphisms also are available [72,73]. The latter have better stability and production efficiency than liposomes [48]. Lipid nanocarriers are better than polymeric nanoparticles (NPs) in terms of biocompatibility and lower toxicity, production cost and scalability, and encapsulation efficiency of highly lipophilic actives [74,75]. Lipid nanocarriers such as solid lipid nanoparticles (SLN) [76], nanostructured lipid carriers (NLC) [77], lipid nanocapsules (LNC) [78], and drug–lipid conjugates [79] are used for various administration routes (i.e., parenteral, oral, and topical ones) [80]. Lipid polymer hybrid nanoparticles (LPHNs) can also be used in the area of bioimaging agents for medicinal diagnostics as delivery vehicles like iron oxide, quantum dots (QDs) fluorescent dyes, and inorganic nanocrystals [81].

#### 2.1.2. Liposomes 

**Figure 3:**
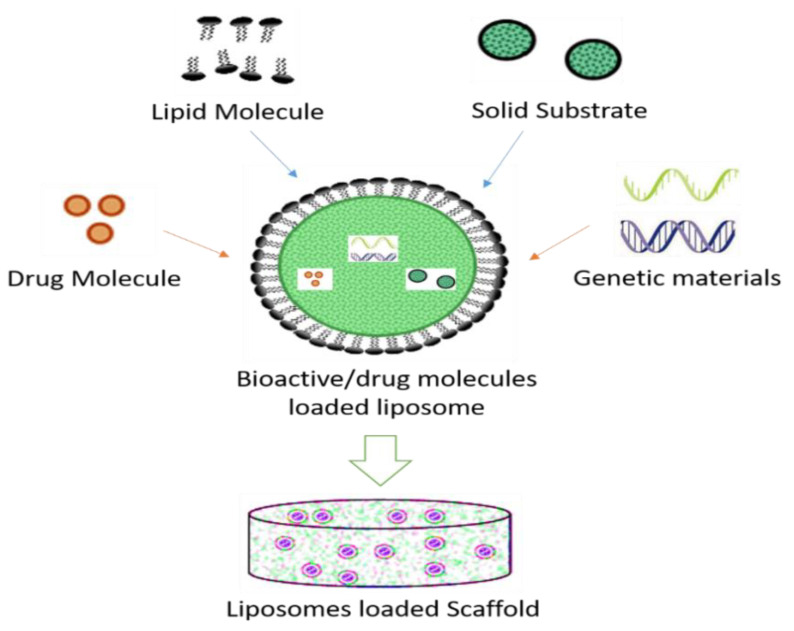
Fabrication of liposome loaded scaffold.

Liposomes were discovered in the mid-1960s by A. D. Bangham [82]. The vesicle of the liposome is easily fabricated in a laboratory and made of one or more phospholipid bilayers [83] (Figure 3). These are self-assembled versatile particles with diameters ranging from nanometer to micrometer scale [84]. Resembling lipid cell membranes, the nature of phospholipid depends on the length of fatty acid chains [48]. They have the ability to encapsulate and carry hydrophobic aqueous agents [82]. They exhibit many advantages over other carrier systems [85,86]. 

Bone morphogenetic protein-2 (BMP-2) is one of the most potent proteins in bone regeneration [87]. For this reason, encapsulation of BMP-2 in nanomaterials has attracted great interest. BMP-2-loaded liposomal-based scaffolds may possess better osteoinductivity and bone formation ability [88]. 

**Figure 4:**
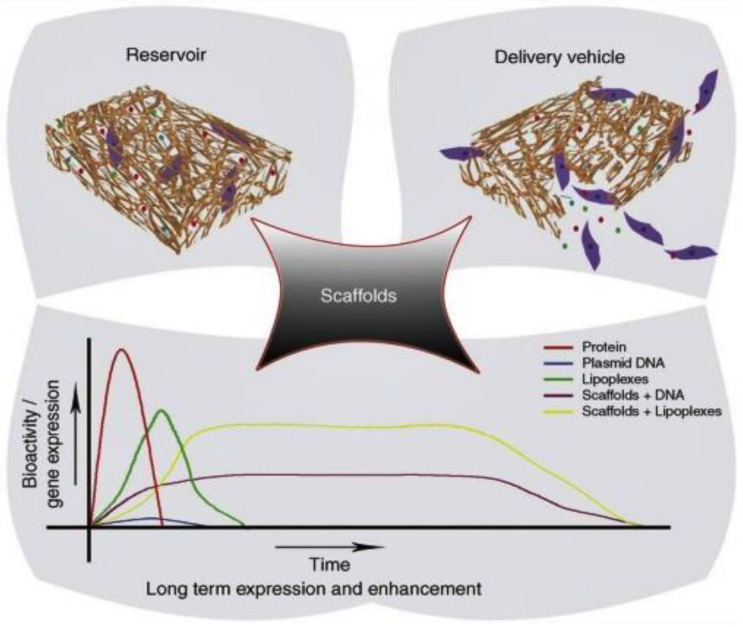
Schematic depiction of the role of tissue-engineered scaffolds in gene delivery [96] (adapted with permission from Elsevier © 2009).

Liposomes can carry drugs directly to the site of action and sustain their levels without causing toxicity for long periods [89]. By changing the composition of lipids, liposome properties can change. Some liposome preparations for anticancer drugs have successfully released on the market by acquiring FDA’s approval [83]. Gentamycin- and vancomycin-integrated liposome-loaded particles are employed for manufacturing of scaffolds [90]. The integration of bioactive aspirin into a liposome delivery system would have a beneficial impact on stem cell osteoblast differentiation [91]. The initial drug amount and the chemical and physical drug properties are considerable factors for the encapsulation efficiency [92]. DOXIL^®^, the first FDA-approved nanodrug, which consists of liposomes encapsulating doxorubicin, was prepared by this remote loading method [93]. This method can also be used for preparing liposomes encapsulating other drugs such as daunorubicin and vincristine [94]. Liposomal systems are highly used despite being the oldest of the non-viralgene-delivery vehicles [95]. Scaffolds used as delivery vehicles for bioactive agents offer many advantages such as enhanced and extended gene expression, and the ability to control a localized delivery of cargo [96] (see Figure 3 and Figure 4).

#### 2.1.3. Dendrimers

Dendrimers are the newest class of highly-defined macromolecules, which differs from simple polymers by branching at each repeating unit [97]. Their step-by-step controlled synthesis is used worldwide for molecular chemistry, while their repeating structure made of monomers relate them to the world of polymers [98,99]. The repetitively branched nanometer-scale dimension of dendrimers is an ideal candidate for a variety of tissue engineering [100], molecular imaging [101], and drug delivery [102] applications. Dendrimers can be a main component of scaffolds mimicking cross-linkers, chemical surface modifiers, and charge modifiers, as well as natural extracellular matrices [103]. 

The combination of dendrimers with other conventional structural polymers, such as proteins, carbohydrates and linear synthetic polymers, leads to obtain new physical, mechanical and biochemical properties of hybrid structures [100,104]. The center of dendrimer may be composed of polypropylimine (PPI), di-aminobutyl (DAB), polyamidoamine (PAMAM), and ethylenediamine (EDA), along with various surface residues such as amine, carboxyl, and alcoholic groups [105]. A dendrimer can be synthesized for particular use in different parts with controlled properties like solubility and thermal stability [106]. 

**Figure 5:**
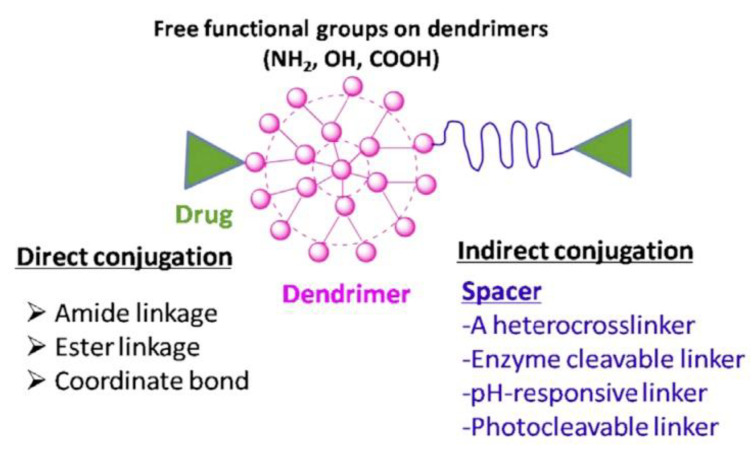
Structure of a typical dendrimer–drug conjugate (Reused with permission from Elsevier [108].)

Dendrimer–drug conjugation is a better approach to the encapsulation of cytotoxic pharmaceuticals. In this way, numerous cytotoxic and anticancer drugs, and targeted individuals such as monoclonal antibodies, peptides, and folic acid, can be conjugated to a single dendrimer molecule [107]. The drug is covalently conjugated to the dendrimer rather than complexed (Figure 5) [108] and these conjugates are relatively more stable.

Dendrimers are a good choice for hydrophobic moieties and poorly water-soluble drugs [109]. PAMAM dendrimer/DNA complexes were employed to encapsulate functional fast biodegradable polymer films used for substrate-mediated gene delivery [110]. 

The physicochemical characteristics, such as solubility and pharmacokinetics, of dendrimers are better than those of linear polymers. Therefore, dendrimers are ideal candidates for incorporation into scaffolds used for tissue engineering applications [111,112]. A few scaffolds were fabricated with dendrimers such as poly(caprolactone) chains conjugated to a poly(L-lysine) dendritic core to fabricate an HA-composite [113], linear PCL/n-HA hybrids [114], N-hydroxy succinimide/1-ethyl-3-(3-dimethyl aminopropyl) carbodiimide (NHS/EDC) cross-linked scaffold [115], and dexamethasone carboxymethyl chitosan/PAMAM [116] for in vitro bone regeneration.

#### 2.1.4. Polymeric Nanomaterials

Polymeric nanoparticles of size range 10 nm to 1 μm are the most advanced noninvasive approaches to tissue engineering and drug delivery applications [117]. They are comprised of repeating units of chain-like macromolecules with multiple structures and compositions [118]. In general, polymeric nanoparticles can be used for different applications by changing the physicochemical properties of nanoparticles. Polymers are differently processed to produce nanofibers [119], spherical nanoparticles [120] and polymeric micelles [121] for specific applications. 

There are several techniques to synthesize polymer-based nanoparticles, applied in tissue engineering [122]. Gelation [123], emulsion–solvent evaporation [124], nanoprecipitation [125], salting-out [122], and desolvation process [126] are generally preferred for natural polymers, like proteins and polysaccharides. Similar to other nanoparticle systems, polymer-based nanoparticles or nanocomposites can be functionalized to perform active targeting [127]. 

**Figure 6:**
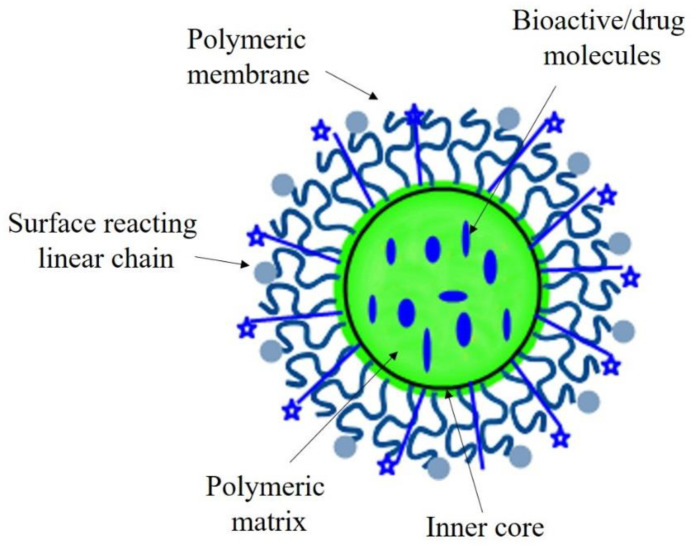
Bioactive/drug molecules loaded polymeric nanoparticles.

Polymeric nanoparticles alter and may enhance the pharmacokinetic and pharmacodynamic properties used for various drug types because they show controlled and sustained release properties [128]. They offer a variety of benefits ranging from the administration of non-soluble drugs to protection of unstable compounds [129]. These nanoparticles can be loaded with therapeutic or bioactive molecules (Figure 6) either by dispersion or adsorption within the polymer matrix, or encapsulation [130,131]. 

Drug release may occur directly from nanoparticles through diffusion and polymeric nanoparticles may dissociate into monomers [132]. Polymers used for nanoparticle fabrication should be degradable via enzymatic or non-enzymatic routes under common metabolic pathways [133,134]. Drug-containing polymeric nanoparticles must be stable during migration to the plasma, that is, at almost neutral pH [135].

Chitosan, collagen, gelatin, hyaluronic acid, alginate, and albumin are representative examples of natural biopolymers [136,137]. Polymeric nanoparticles are one of the fastest-growing platforms for the applications in tissue engineering because of their biocompatibility, biodegradability, low cytotoxicity, high permeation, ability to deliver poorly soluble drugs, and retaining bioactivity after degradation [117]. Some newly designed polymeric nanoparticles are sensitive to pH, temperature, oxidizing/reducing agents, and magnetic field which support a high efficiency and specificity for tissue engineering applications [138,139]. Due to good biocompatibility and adjustable chemical composition, and their ability to reorganize, polymeric nanoparticles are very promising as nanobiomaterials for the fabrication of scaffolds or bone substitutes [140]. Plasma protein-based nanoparticles have shown high biodegradability, bioavailability, long in vivo half-lives, and long shelf lives without any toxicity. Blood plasma is a complex mixture of 100,000 proteins, but only two of these proteins have been used in drug administration and tissue regeneration [141,142].

##### Chitosan

**Figure 7:**
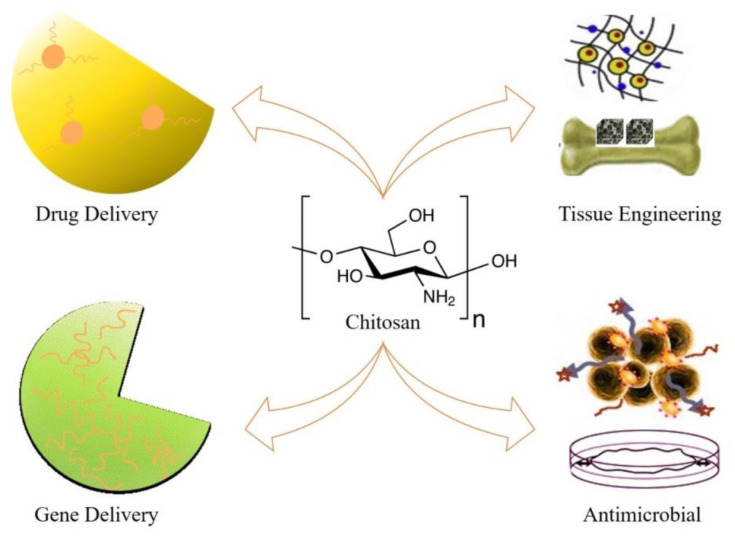
Prospective applications of chitosan.

Chitosan is a natural and nontoxic linear biopolymer synthesized from alkaline N-deacetylation of chitin [143]. It can be extracted from exoskeleton of crustacean shells (i.e., crabs and shrimps) some microbes, yeast, and fungi [144]. It has different molecular weights and is soluble in various organic solutions at pH 6.5 and below. The shape of the chitosan nanoparticles is affected by the degree of deacetylation [145,146]. The presence of amine and hydroxyl group leads the use of these compounds in many research areas [147,148]. Chitosan has outstanding biochemical properties, making it very attractive for applications in many areas including tissue engineering and/or regenerative medicine (Figure 7) [149]. Chitosan nanoparticles carry well therapeutic agents and biomolecules because of their high biocompatibility and biodegradability. Because of their small size, they can pass through biological barriers in vivo and deliver the drugs at the targeted site [150].

Applications of Chitosan: Scaffolds prepared from chitosan and ceramics, especially hydroxyapatite, may have superior osteoconductive properties [151]. Bone morphogenetic protein-2 (BMP-2)-loaded chitosan nanoparticles used for the coating of Ti implants were selected in order to examine bone regeneration in mice [55]. Chitosan and growth factor (BMP-7) were used to functionalize a thick electrospun poly(ε-caprolactone) nanofibrous implant (from 700 μm to 1 cm thick), which produced a fish scale-like chitosan/BMP-7 nano-reservoir. This nanofibrous implant mimicked the extracellular matrix and enabled in vitro colonization and bone regeneration [152]. There, the polycationic nature of chitosan entails an antimicrobial behavior at nanoscale [153]. Besides the orthodontic field, there are relevant applications of chitosan in skin healing, nerve regeneration, and oral mucosa [39]. Nanobioglass incorporated chitosan-gelatin scaffolds showed excellent cytocompatibility and ability to accelerate the crystallization of bone-like apatite in vitro [154,155]. The nanocomposite of chitosan/hydroxyapatite-zinc oxide (CTS/HAp-ZnO) supporting organically modified montmorillonite clay (OMMT) was synthesized and used for hard tissue engineering applications [156]. BMP-2 and BMP-7 loaded poly(3-hydroxybutyrate-co3-hydroxyvalerate) nanocapsules were used for the fabrication of chitosan-poly(ethylene oxide) scaffolds [157]. Mili et al. [158] used nerve growth factor (NGF) loaded chitosan nanoparticles for neural differentiation of canine mesenchymal stem cells. Freeze-dried nano-TiO_2_/chitosan scaffolds showed high biocompatibility and antibacterial effects [159]. Chitosan-poly(vinyl alcohol)-gum tragacanth (CS/PVA/GT) hybrid nanofibrous scaffolds showed 20 MPa ultimate tensile strength and supported L929 fibroblast cells growth [160]. Collagen–chitosan–calcium phosphate microsphere scaffolds fused with glycolic acid did not show relevant differences in their degradation, cytocompatibility, porosity, and Young’s modulus [160,161].

##### Collagen

**Figure 8:**
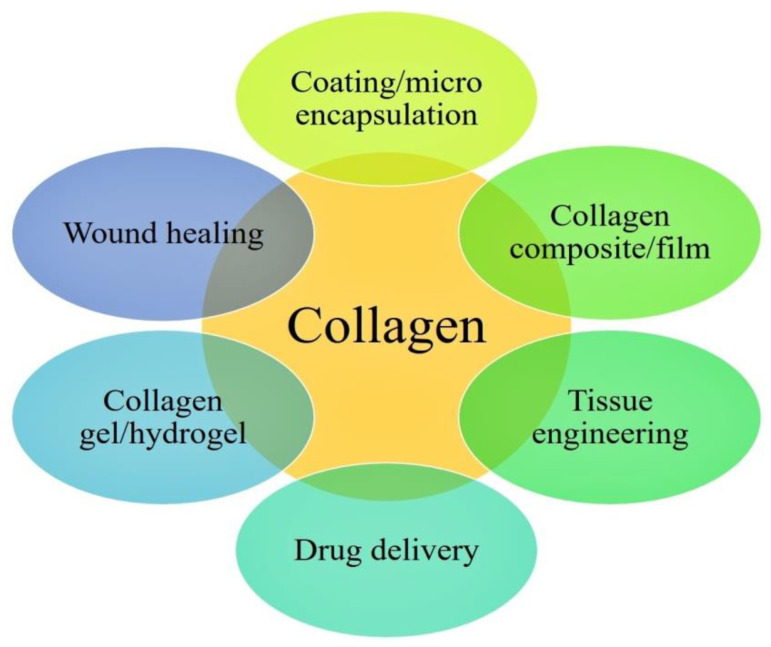
Biomedical applications of collagen.

The main constituents of living human bone are collagen type-1 (protein) and calcium phosphate or hydroxyapatite (mineral) [162]. Collagen is the major structural protein of the soft and hard tissues in living organisms [163]. It can have a significant role in preserving biological and structural integrity of extracellular matrix (ECM) [164]. It is a versatile material that is widely used in the biomedical field (Figure 8) due to advantages including high biocompatibility and biodegradability [165]. Collagen is mainly used as a carrier for drug delivery as well as osteogenic and bone filling material [166]. Collagen matrix was also used to deliver gene promoting bone synthesis [167]. Collagen with recombinant human bone morphogenetic protein-2 was used to monitor bone formation [168].

Bone morphogenic protein (BMP)-loaded collagen activates osteoinduction in the host tissue [169]. Collagen-based nanospheres/nanoparticles can be used as a systematic delivery carrier for various therapeutic agents or biomolecules [166]. As collagen type-I and hydroxyapatite are a basic part of the bone, hydroxyapatite and collagen were used to fabricate scaffolds that enhance osteoblast differentiation and accelerate osteogenesis [170]. 

Collagen-based biomaterials in various formats such as 3-D scaffolds have been employed for tissue engineering [171]. The combination of collagen with elastin was successfully fabricated and in vitro tests proved the adhesion and proliferation of cells without any cytotoxicity [172]. Collagen-based inks were used for 3D bioprinting employed for tissue repairing and scaffold fabrication. The collagen-based ink was extruded with a temperature stage of −40 °C, followed by freeze-drying and cross-linking by using 1-ethyl-(3-3-dimethylaminopropyl) hydrochloride solution [173].

##### Gelatin

**Figure 9:**
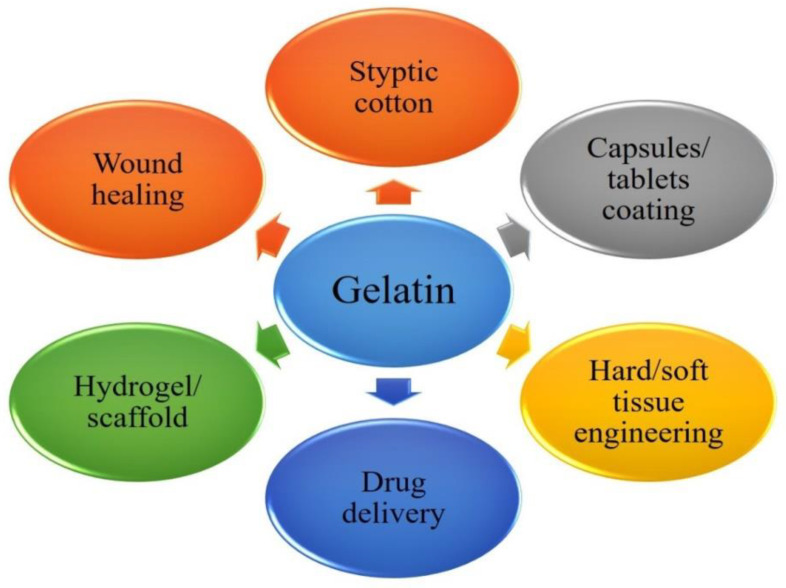
Biomedical and pharmaceutical uses of gelatin.

Gelatin represents a derivative of collagen, extracted by collagen hydrolysis from the skin, bones, and/or connective tissues of animals. It is a cost effective, biocompatible, and biodegradable polymer, which supports cross-linking of functional groups. Gelatin is a versatile polymer that is known for his wealth merits [174]. Pharmaceutical or medical grade gelatin has fragility and transparency for tablet coatings, suspensions, capsule formulations, and nano-formulations (Figure 9). 

Because of their great biocompatibility, the injected gelatin-loaded nanoparticles have been reported in the skeletal system [24,175,176]. It is a polyampholyte at pH 9 (gelatin A) and pH 5 (gelatin B). Gelatin nanoparticles are used as a biomaterial for the delivery of biomolecules and therapeutic agents [177]. However, digestive process of gelatin showed low antigenicity, with the formation of harmless metabolic products. In order to prevent infectious disease transmission, genetic engineering approaches were used for the production of human recombinant gelatin [178,179]. 

At the nanoscale, gelatin shows high biocompatibility, biodegradability, and low immunogenicity [180]. The presence of a higher number of functional groups on polymer backbone helps with crosslinking and chemical modification [181]. The cross-linking is necessary to stabilize the macromolecular structure of gelatin is not stable at normal body temperature due to the low melting temperature [182,183]. 

Gelatin methacryloyl (GelMA) hybrid hydrogel demonstrated a wide range of tissue engineering applications. When exposed to light irradiation, GelMA scaffolds convert into hydrogels with tunable mechanical properties [184]. Gelatin enables therapeutic cell adhesion without comprising cell phenotypes [185]. Porous HA-gelatin microparticles (1 to 100 μm) support human osteoblast-like Saos-2 cells growth and cell delivery [186]. A mechanically strong gelatin–silk hydrogel composite was prepared by direct blending of gelatin with amorphous Bombyxmori silk fibroin (SF) [187]. Gelatin coated polyamide (PA) scaffold showed good biomechanical, cell attachment, and wound healing characteristics while being transplanted to nude rats [188]. Poly(lactide-co-glycolide) (PLGA)–gelatin fibrous scaffolds possess the highest Young’s modulus (770 ± 131 kPa) and tensile strength (130 ± 7 kPa) [189]. Methacrylamide-modified gelatin (GelMOD) 3D CAD scaffolds showed excellent stability in culture medium and support porcine mesenchymal stem cell adhesion and subsequent proliferation [190].

**Figure 10:**
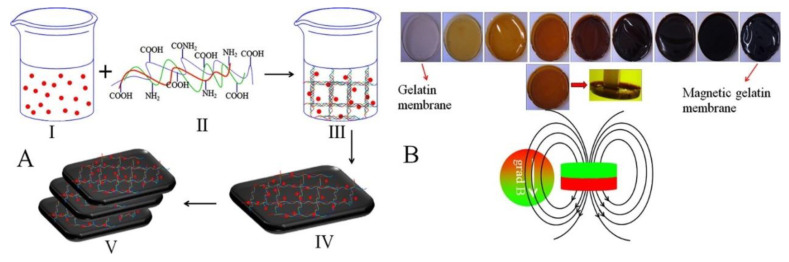
(**A**) Fabrication of a multilayered magnetic gelatin scaffold. (**B**) Magnetic gelatin membranes with increasing MNPs concentration from left to right as well as a representation of the properties of the magnetic gradient. (Adapted with permission from © 2015 American Chemical Society [192]).

Gelatin-based microcarriers used embryonic stem cell delivery for the applications in tissue engineering [191]. The magnetic nanoparticles were assembled with magnetic gelatin membranes to produce 3D multilayered scaffolds (Figure 10), which are used for controlled distribution of magnetically labeled stem cells [192]. 

##### Poly (Lactic-co-glycolic) acid (PLGA)

PLGA is considered as one of the most efficient tissue engineering materials due to its (i) high biocompatibility, (ii) biodegradability, (iii) potential to interact with biological materials, and (iv) clinical use approved by FDA [193]. Biodegradable biomolecule-loaded PLGA nanoparticles can be used for the preparation of a drug delivery system, which can be further utilized in scaffold fabrications [194,195,196]. These nanoparticles may increase the mechanical properties of the scaffolds but decrease swelling behavior without changing the morphology of the scaffold [197]. Afterward, this system is effective to prepare a controlled release platform for model drugs that favors the bio-distribution and development of clinically relevant therapies [198]. Different methods such as gas foaming [199], porogen leaching [200], solid freedom fabrication [201], and phase separation [202] can be used for PLGA scaffolds fabrication.

#### 2.1.5. Carbon Nanostructures

**Figure 11:**
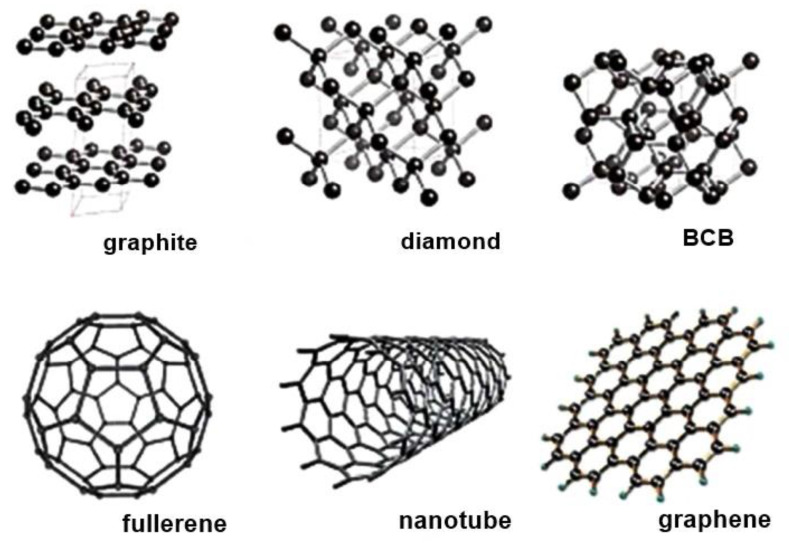
Structure of various allotropes of carbon (adapted with permission from Royal Society of Chemistry [205]). In the figure, “BCB” stands for “benzocyclobutene”.

Carbon nanomaterials are great candidate materials for bone tissue engineering due to their conductivity, lightweight, stability and strength [203]. Nanostructures such as fullerenes, carbon nanotubes, carbon nanofibers, and graphene are the most common structures (Figure 11) [204,205].

Han et al. [205] pointed out that carbon is biocompatible and can be used in many clinical applications, such as prosthetic heart valves. However, a pure form of carbon nanomaterials cannot be used as a substrate for bone tissue [206]. Therefore, carbon-based materials are used in combined form to fabricate scaffolds [207]. Carbon nanostructures doped or reinforced compositions became more popular due to their high performance and compatibility with bone tissues [203].

Carbon nanofibers (CNF) are cylindrical or conical structures of various diameters and lengths. The interior structure of the CNF contains an improved layout of graphene sheets. Graphene is a single-layer two-dimensional material composed of long-edged reactive carbon atoms. Graphene leaves are characterized by stable dispersion and orientation of nanofillers [208,209,210].

Carbon nanotubes (CNT) enhance mechanical and electrical properties, which helps to generate innovative products. CNTs are one of the ideal and favorable materials used for designing novel polymer composites [211]. Many authors focused on the progress of composite materials fabrication-integrating CNTs to enhance its applications in biomedical field [212,213,214,215,216]. 

Nanodiamonds (4–10 nm) are typically different from other nanostructures as they are sp^3^ hybridized [203]. They show admirable protein binding ability and can be used as a carrier for some biomolecules such as BMP-2 [217]. The carbon nanotube/gold hybrids are employed commonly for the delivery of the anticancer drug doxorubicin hydrochloride into A549 lung cancer cell line [218]. 

Nanoscaffolds can be produced by electrospinning poly(ε-caprolactone) (PCL) and different types of carbon nanomaterials such as carbon nanotubes, graphene, and fullerene [219]. Mesoporous silica (mSiO_2_) decorated carbon nanotubes (CNTs) hybrid composite were used for the simultaneous applications of gentamicin and protein cytochrome C delivery and imaging [220]. Single-walled carbon nano-horns encapsulated with positively charged lipids complex were used for targeted drug and protein delivery [221].

#### 2.1.6. Summary and Statistical Analysis of the Survey on Organic Nanobiomaterials 

The survey on organic nanobiomaterials presented in Section 2.1 regarded some 200 articles. Polymeric nanomaterials, carbon nanostructures, and nanocomposite materials are the most widely investigated subject (60.8% of the studies), followed by dendrimers (21.1%) and lipids/liposomes (18.1%). While dendrimers and lipid/liposomes are mainly utilized as nanocarriers, the other nanomaterials cover a much broader spectrum of applications. The development of new nanomaterials (especially carbon nanomaterials or materials including natural bone constituents such as, for example, collagen) that can improve tissue regeneration, cell growth, and drug/protein delivery currently represents the main research area in the field of organic nanobiomaterials with a strong tendency to design hybrid materials and improve fabrication techniques of the resulting nanocomposite materials/scaffolds/structures. Such a trend has become very clear in the last 5–6 years. However, much work remains to be done in order to fully understand interactions between different phases of nanocomposite materials and cell/tissues to be repaired/treated. Another important issue strictly related to the above mentioned one is how to “optimize” the composition of the nanocomposite for the specific purposes on which the material itself is designed.

### 2.2. Inorganic Nanobiomaterials

Inorganic biomaterials are those lacking carbon element and they are widely employed for in vivo and in vitro biomedical research [222]. These crystalline or glass structured nanomaterials are used to replace or restore a body tissue [36]. The main applications of inorganic biomaterials, including bioceramics and bioglasses, are for orthopedics and dentistry. Modifications in composition and fabrication techniques may produce a range of biocompatible materials such as bioceramics [223]. Natural bone also includes inorganic materials like calcium (Ca) and phosphorus (P) in the form of hydroxyapatite (HA) crystals, as well as carbonate (CO_3_^2−^), potassium (K), fluoride (F), chlorine (Cl), sodium (Na), magnesium (Mg), and some trace elements including copper (Cu), zinc (Zn), strontium (Sr), iron (Fe), and silicon (Si) [224]. Therefore, it is very logical to investigate on nanomaterials based on these inorganic constituents.

Table 2 presents a general classification of inorganic nanobiomaterials and summarizes representative applications of each material in tissue engineering. The following subsections present a general description of each nanomaterial type listed in Table 2 and a detailed literature survey on the corresponding developments for tissue engineering.

#### 2.2.1. Nano Silica

A huge amount of investigations on biomedical applications of silica nanostructures have been carried out in the past decade [249]. The ability to synthesize uniform, porous and dispersible nanoparticles, together with the fact that particles’ size and shape can be easily controlled [226], certainly favored the variety of applications of silica in tissue engineering [250]. Furthermore, as silica is biocompatible and chemically stable [251,252], it has been used also for biomedical imaging and medication administration [225], either itself or as a coating of other compounds [251].

**Figure 12:**
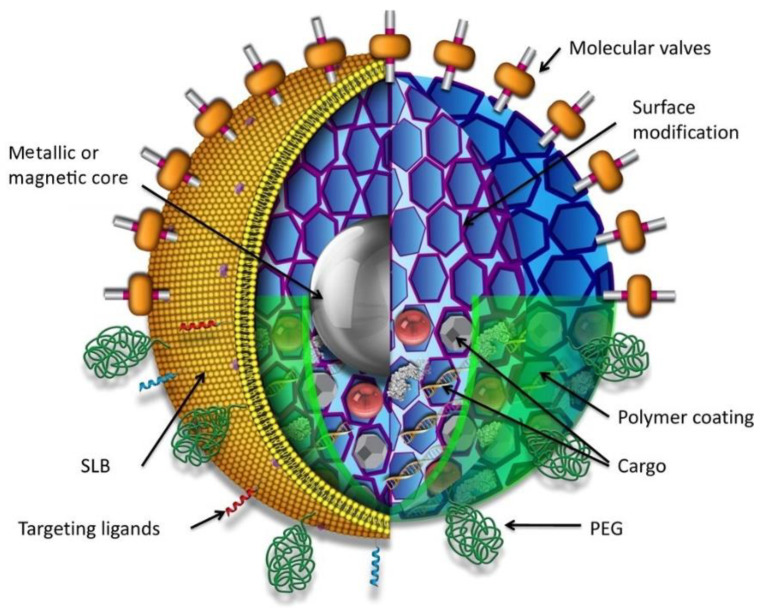
Schematic of a multifunctional mesoporous silica nanoparticle showing possible core/shell design, surface modifications, and multiple types of cargos. (Adapted with permission from © 2013 American Chemical Society [250]).

Mesoporous silica nanoparticles (MSNPs) have been used as a drug delivery vehicle [253] and to improve mechanical properties of biological materials. It was noted their use as well as for sustained and prolonged release or administration of intracellular genes in bone tissue engineering [226]. MSNPs work as efficient biocompatible nanocarriers due to (i) high visibility, (ii) dispersibility, (iii) binding capability to a target tissue, (iv) ability to load and deliver large concentrations of cargos, and (v) triggered or controlled release of cargos [250]. The functioning of MSNPs can be tailored by modifying the silanol group present within the pore interiors and on the outer surface. These positive chemical moieties are adsorbed by negatively charged SiO^–^ groups at neutral pH, through electrostatic interactions (Figure 12). 

Anitha et al. [254] reported a composite matrix containing crystalline rod-shaped core with uniform amorphous silica sheath (Si–n HA), which showed good biocompatibility, osteogenic differentiation, vascularization, and bone regeneration potential. Silicate containing hydroxyapatite stimulates cell viability of human mesenchymal stem cells for extended proliferation [255]. Zhou et al. [256] synthesized PLGA–SBA15 composite membranes with different silica contents by electrospinning method; these membranes showed better osteogenic initiation then the pure PLGA membranes. Ding et al. [257] successfully fabricated levofloxacin (LFX)-loaded polyhydroxybutyrate/poly(ε-caprolactone) (PHB/PCL) and PHB/PCL/sol–gel-derived silica (SGS) scaffolds, which support the growth of MG-63 osteoblasts. A microfluidic device was used to generate photo-cross-linkable gelatin microgels (GelMA), coupled with providing a protective silica hydrogel layer for applications in injectable tissue constructs [258]. Dexamethasone (DEX)-loaded aminated mesoporous silica nanoparticles (MSNs-NH2) were prepared via electrophoretic deposition (EPD) and successfully incorporated within poly(l-lactic acid)/poly(ε-caprolactone) (PLLA/PCL) matrix to fabricate composite nanofibrous scaffolds for bone tissue engineering applications [259]. 

#### 2.2.2. Nano Bioglass

**Figure 13:**
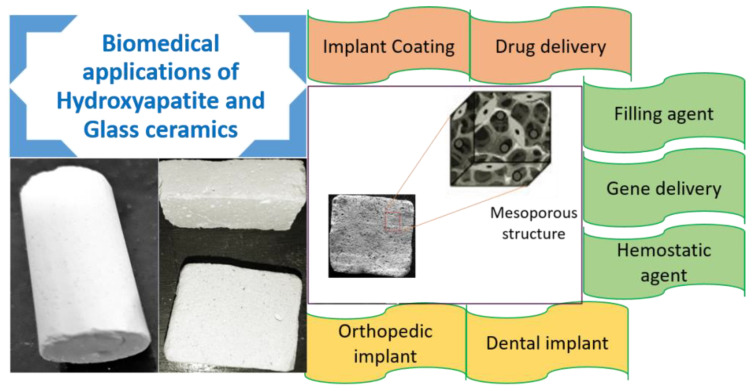
Biomedical applications of hydroxyapatite and glass ceramics.

Bioglasses (BG) have been intensively investigated as biomaterials since their discovery in 1969 and first developments in the 1970s made by L. Hench [260]. Compared to common glass, bioglass contains less silica and higher amounts of calcium and phosphorous. As a biomaterial for tissue engineering, bioglass is applied independently or in combination with a number of polymers [261] (Figure 13). BG can arouse fibroblasts with higher bioactivity by accelerating bioactive growth factors and proteins as compared to untreated fibroblasts [262]. 

BG degrade slowly when implanted into the targeted patient’s site and release ions, which favors the biosynthesis of hydroxyapatite [263]. The silica-rich surface of bioglass promotes the exchange of Ca^2+^ and PO_4_^3−^ with physiological fluid, which leads to the generation of a Ca–P layer [264,265]. This biodegradation may be enhanced by the presence of a SiO_2_ network, which forms non-bridging silicon-oxygen bonds [266]; the low connectivity of the SiO_2_ network enhances dissolution of bioglass while the presence of Na and Ca forms Si–O–Si bonds and reduces dissolution rate. Mesoporous BG can be fabricated using the sol-gel method, which can be a good carrier for targeted drug delivery [267]. The sol–gel method was also used by Kumar et al. [268] to develop bioglass nanoparticles with a higher content of silica, which are suited for bone tissue applications.

**Figure 14:**
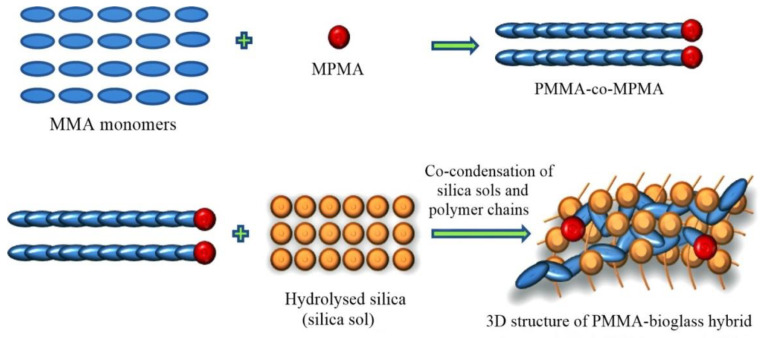
Schematic procedure for the fabrication of a PMMA-bioglass class II hybrid (Adapted with permission from © 2013 American Chemical Society [272]).

Bioglass nanoparticles show high biocompatibility and surface area, which can enhance in vitro osteoconductivity as compared to layer and microsized particles of bioglass [269]. The size of the particles can be modified by changing the synthesis parameters and techniques. However, because of its brittleness, the glass alone cannot be used to heal large bone defects [270]. In order to solve this issue, Bioglass 45S5 was used with poly(D,L-lactide) (PDLLA), a biodegradable polymer, to form a composite scaffold with enhanced biomechanical characteristics [271]. The early failure of a bioglass composite at the interface occurs because of nonuniform mechanical strength, phase separation, nonhomogeneous mixture, and different degradation properties of two compounds. A hybrid composite of poly(methyl methacrylate) (PMMA) and bioactive glass was manufactured via the sol-gel method (Figure 14) to enhance physicochemical and mechanical properties [272].

An elastin-like polypeptidic and bioglass (ELP/BG) hydrogel was also fabricated that is mechanically robust, injectable, and self-healable. This ELP/BG biocomposite can be useful for drug delivery and tissue engineering purposes [273]. A 3D construct of type-I collagen and 45S5 Bioglass meets the basic requirements of a scaffold including biocompatibility, osteoconductivity, osteoinductivity, and biodegradability [274]. Bioglass nanoparticles were also used with bacterially derived poly(3-hydroxybutyrate) to fabricate bioactive composite film using a fermentation technique [275]. Different glass modifiers (Mg^2+^, Ca^2+^, and Sr^2+^) were used to prepare borosilicate bioactive glasses through a melt-quenching technique which showed good antibacterial properties [276]. Poly(propylene fumarate) (PPF) was used to functionalize bioglass particles that enhance the bioactivity and cell adhesion, proliferation, and bone regeneration [277].

#### 2.2.3. Nano Hydroxyapatite

Hydroxyapatite (Ca_10_(PO_4_)_6_(OH)_2_) is a significant natural mineral constituent of bones (70% wt.) and teeth (96% wt.) [278,279]. Synthetic HA is a biocompatible ceramic material, used for biomedical applications (Figure 13) because it may replicate the behavior of mineral part of the bone [280,281]. It shows outstanding biocompatibility with bones, teeth, skin, and muscles, both in vitro and in vivo [282,283]. The stoichiometric molar ratio Ca/P in synthetic HA of 1.67 is not the actual ratio in the hydroxyapatite of normal bones, because of the presence of other elements such as C, N, Fe, Mg, and Na [284]. Hydroxyapatite (HA) can be easily synthesized by using different methods such as hydrothermal, sol–gel, and co-precipitation methods [285]. The comparison of mineral compositions of hydroxyapatite, bone and teeth is shown in Table 3 [286,287]. 

HA shows such excellent biocompatibility, bio-inertia and bioactivity without toxicity, immunogenicity [288,289]. It has a good ability to make bonds with bone directly and it is primarily used in therapeutic applications such as implants and fillers for bones and teeth in different forms [290]. To overcome the low mechanical strength of hydroxyapatite scaffolds, a large number of natural and synthetic polymers were combined with HA such as collagen, polyethylene, polylactic acid, alginates, poly(methyl methacrylate), and polycaprolactone [136].

**Figure 15:**
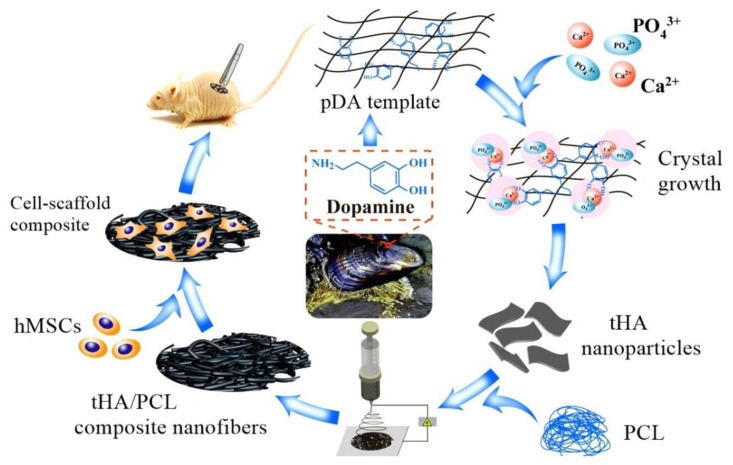
Schematic illustration of preparation and evaluation of tHA/PCL composite nanofibers. (Adapted with permission from © 2016 American Chemical Society [292].)

Woodard et al. [291] compared the activity of nano- and microsized ceramic materials in the body. Their studies demonstrated a substantial increase in osteoblast adhesion and protein adsorption in nanomaterials. The major components of the inorganic nanostructure can have a higher biological activity than micro-components [245]. Polydopamine (pDA)-templated hydroxyapatite (tHA) was introduced into polycaprolactone (PCL) matrix to make bioactive tHA/PCL composite based fibrous scaffold; in vitro and in vivo investigations (Figure 15) showed a favorable cytocompatibility at a given concentration of tHA (0–10% wt.) [292]. 

A new type of scaffold with bamboo fiber (5%) incorporated nano-hydroxyapatite/poly(lactic-co-glycolic) (30%) was fabricated via freeze-drying; bamboo fibers improved biomechanical properties of n-HA/PLGA composite scaffolds thus developing a superior potential for bone tissue engineering [293]. Sol–gel synthesized hydroxyapatite–TiO_2_-based nanocomposites synthesized in supercritical CO_2_ have better Young’s and flexural moduli than PCL/HAp composites [294]. A set of techniques including molding/particle leaching and plasma-treated surface deposition were used to fabricate bilayered PLGA/PLGA-HAp composite scaffold [295]; the in vivo rat model experiment proved that the new composite is suitable for osteochondral tissue engineering applications. Electrospinning mediated poly(ε-caprolactone)−poly(ethylene glycol)−poly(ε-caprolactone) (PCL–PEG–PCL, PCEC) and nano-hydroxyapatite (n-HA) composite scaffolds showed good biocompatibility and nontoxicity [296]. Hydroxyapatite/Na(Y/Gd)F_4_:Yb^3+^, Er^3+^ composite fibers [297], and gadolinium-doped mesoporous strontium hydroxyapatite nanorods [298] were successfully used in drug storage/release applications.

#### 2.2.4. Silver Nanoparticles

**Figure 16:**
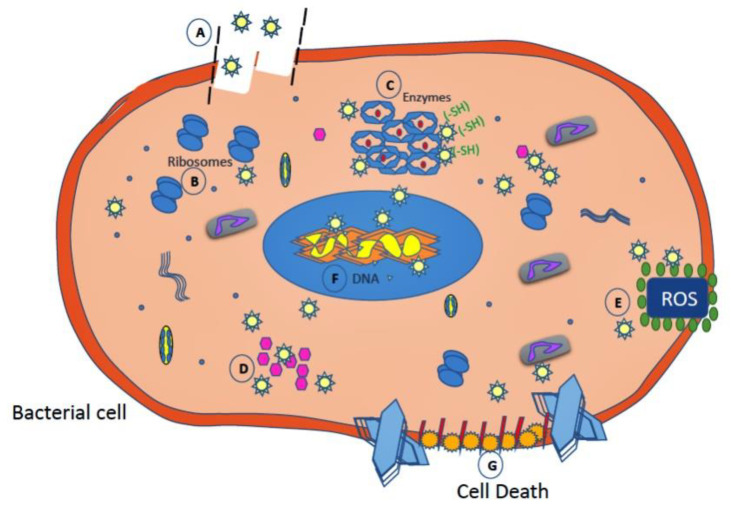
Schematic diagram representing mechanisms of action of Ag nanoparticles for antibacterial action (adapted with permission from Elsevier © 2018 [303]). (**A**) AgNP diffusion and uptake into the bacterial cell. (**B**) Destabilization of ribosomes. (**C**) Enzyme interaction. (**D**) Interruption of electron transfer chain. (**E**) Reactive oxygen species (ROS). (**F**) DNA damage. (**G**) Cell death.

Silver proved its bactericidal activities against many bacteria since 1000 B.C. Now silver is used as an antiseptic, antibacterial, and antitumor agent [299]. Because of their strong antibacterial activity against both Gram-positive and Gram-negative bacterial strains, silver nanoparticles were widely used for fabricating antibacterial nanocomposite-based scaffolds and coated implants [235,237]. Furthermore, silver may be combined with different materials such as CNT [300], chitosan, HA [301], and manganite [302] to get a specific function. Ag-doped or coated implants allow reducing the number of bacterial infections without interfering with bone cell growth in the body (Figure 16 [303]). The antimicrobial activity of Ag has been reported against *Escherichia coli* [304], *Candida albicans* [11], *Vibrio cholera* [305], and *Staphylococcus aureus* [306]. 

In addition to antibacterial activity, Ag nanoparticles promote wound healing, reduce scar formation, and reduce inflammation [307]. Silver nanoparticles have many applications as antimicrobial agents when combined with different biological substances [308]. A micrometer-sized surface-enhanced Raman spectroscopy (SERS) substrate, core–shell microparticles composed of solid carbonate core coated with silver nanoparticles, and polyhedral multishell fullerene-like structure were developed for biomedical applications [309]. Soft poly(vinyl alcohol) (PVA) hydrogel films containing silver particles prepared on solid biodegradable poly(l-lactic acid) (PLLA) exhibit both antibacterial and reduced cell adhesion properties [310]. Biocompatible maleimide-coated silver nanoparticles (Ag NPs) can be used as co-cross-linkers for the preparation of a nanocomposite gelatin-based hydrogel. Covalently bound Ag nanoparticles support swelling and drug release properties of composite hydrogel without producing toxicity [311]. In situ fabricated Ag NPs (4-19 nm) and immobilized on titanium by using a plasma immersion ion implantation process motivated osteoblast differentiation in rat bone marrow stem cells (BMSCs) [312]. Patrascu et al. [313] fabricated collagen/hydroxyapatite-silver nanoparticles (COLL/HA-Ag)-based antiseptic composite for biomedical applications. 

#### 2.2.5. Gold Nanoparticles

Gold nanoparticles (GNPs) are defined as a colloid of nanometer-sized particles with better properties than bulk gold. They are produced in different shapes such as spheres [314], rods [315], star-like [316], and cages [317]. GNPs possess unique characteristics, such as easy-to-control, nanoscale size, easy preparation, high surface area, easy functionalization, and excellent biocompatibility, that make them highly suited for many tissue engineering and more in general for biotechnology applications [318,319]. GNPs are definitely superior over other types of nanoparticles in terms of low toxicity and colloidal stability. Furthermore, they present an outstanding physicochemical behavior, which is related to local plasmon resonance phenomena.

Gold nanoparticles were utilized for biosensing [320], bioimaging [321,322,323], cancer therapy [324], gene delivery to enhance osteogenic differentiation [325], and photo-thermal therapy [229,314,316,323]. Gold nanoparticles were also combined with other materials such as silica (to produce core and shell nanoparticles) [318,323] as well as natural polymers (to improve mechanical properties) and synthetic polymers (to enhance biocompatibility) [318]. 

**Figure 17:**
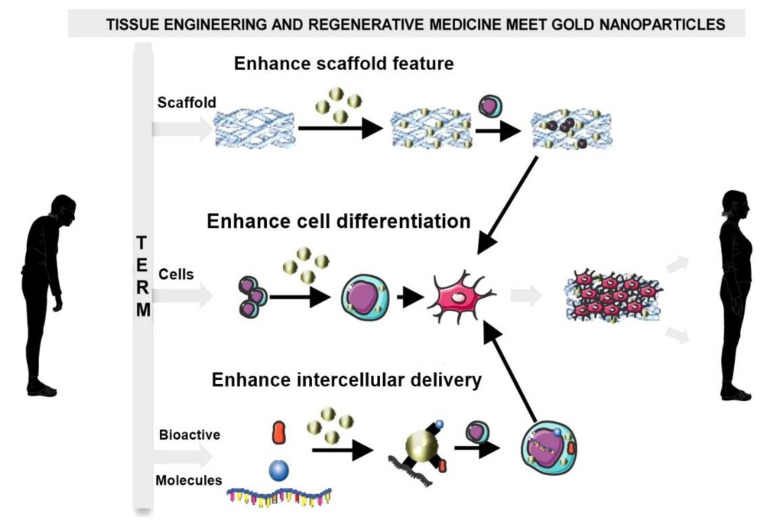
Scheme representing the importance of introducing GNPs in tissue engineering and the regenerative medicine (TERM) realm [326]. (Adapted with permission from Elsevier © 2017).

Due to their excellent biocompatibility and chemical inertness, gold nanoparticles became the ideal choice for the preparation of scaffolds in many cases [318]. The mission of GNPs in tissue engineering and regenerative medicine is to act as a multimodal tool in order to improve scaffold properties, cell differentiation and intracellular growth factor delivery (Figure 17), while monitoring cellular events in real-time [326].

#### 2.2.6. Titanium Dioxide

Titanium is widely used in many surgical applications (e.g., prostheses and implants) because of its excellent biocompatibility, good mechanical properties, and lower mass density than steel [327]. The low density and high specific strength of titanium results in lightweight implants with good mechanical properties [238,239]. Furthermore, the smooth surface of Ti mesh prevents bacterial contamination instead of adsorbate materials. Therefore, titanium mesh provides an excellent solution to guide bone regeneration [243]. 

Nanostructured TiO_2_ materials of various morphologies such as nanoparticles, nanorods, nanowires, nanotubes, and other hierarchical nanostructures can be produced using different techniques such as, for example, microwaves [328,329], hydrothermal/solvothermal processes [330,331], sol–gel [332,333], anode oxidation [334,335], chemical vapor deposition [336,337], sonochemical processes [338,339], and green synthesis [340,341,342]. 

**Figure 18:**
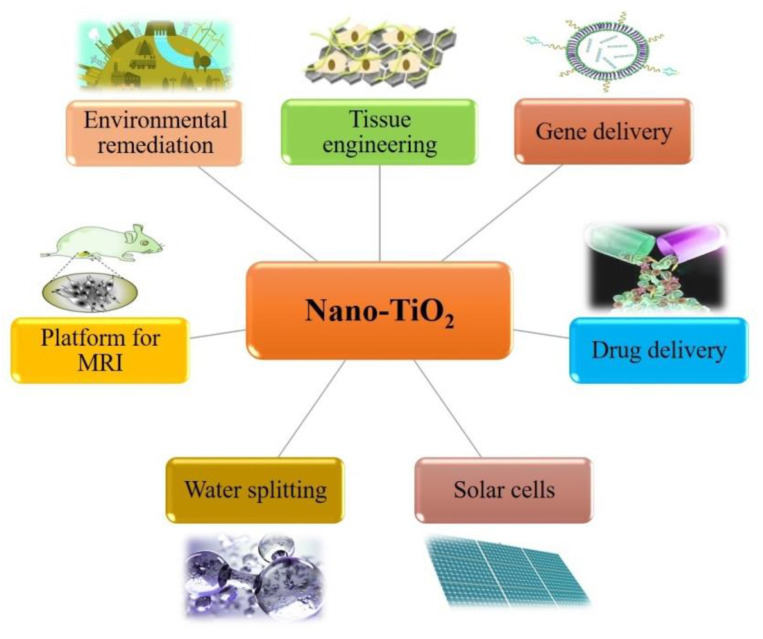
Schematic representation of the many fields of applications of nanostructuredTiO_2_.

As can be seen from Figure 18, nanostructured TiO_2_ is a multifunctional material for a wide range of applications in engineering and biomedical areas. Interestingly, TiO_2_ nanoparticles represent a miniature of electrochemical cells capable of light-induced redox chemistry. This quality can be used for manipulating biomolecules and cell metabolic processes. TiO_2_ nanoparticles prove to have a higher affinity for binding proteins and other cellular components when used within cellular environment [343,344]. TiO_2_ nanoparticles can also be used to enhance photodynamic therapy (PDT) and sonodynamic therapy (SDT) [345].

Titanium nanotubes (TNTs) possess excellent biocompatibility and drug-releasing performance. Furthermore, they can be generated on the surface of the existing medical implants [346,347]. The physical adsorption of the drugs promotes the anti-inflammatory properties of the TNTs, and with improved osteoblast adhesion, the drug-eluting technique is extended [348]. 

TiO_2_ based scaffolds are biocompatible, have good osteoconductive performance and antibacterial properties [349], and show high porosity, excellent interconnectivity, and sufficient mechanical strength [350,351]. Nanostructured TiO_2_ can be combined with several polymers including polylactic acid (PLA) [352]; poly(ether-ether ketone) (PEEK) [353]; poly(lactic-co-glycolic acid) (PLGA) [354]; and inorganic materials such as SiO_2_ [355], Al_2_O_3_ [356], bioglass [357], hydroxyapatite [358], graphene [359], and calcium phosphate [360].

Nano-TiO_2_ surface coated implants can limit autoimmune reactions between the underlying bone tissue surfaces and the implant [361]. However, material deterioration or generation of chronic inflammation in the implanted tissues may reduce success rate [361,362]. Various TiO_2_ nanostructures were used for loading and eluting cefuroxime as an antibiotic on orthopedic implants [363].

#### 2.2.7. Zirconia 

Zirconia was first recognized by M.H. Klaproth in 1789 and used as a pigment for ceramics for a long time [364]. Since the 1970s, zirconia received massive consideration as a biomedical material in association to its chemical and biological inertness [365]. Consequently, zirconia was also used to overcome the brittleness of alumina and the consequent failure of implants [366], and as a material for the repair and replacement of bones due to its unique biomechanical properties [367].

Investigations on zirconia biomaterials began in the 1960s. Classical orthopedics studied for many years have used zirconia in the area of hip replacement [368,369]. Zirconium oxide (zirconia) possesses improved mechanical properties and has become one of the most popular ceramic materials in the field of healthcare due to its high biocompatibility and low toxicity [364,370]. 

Zirconia is one of the most useful structured ceramics because it provides high resistance to bending and fracture. However, zirconium oxide with a low fracture toughness due to the presence of alumina abrasive grains [371] also was introduced as an alternative to having excellent wear resistance due to the unwanted release of orthopedic alumina. Porous zirconia stents can be manufactured by cutting CAD/CAM blocks in the desired shape, and zirconia stents assembled with HA significantly increase the volume of new bone formation in vivo [372]. 

While it might be concluded that zirconia has one of the best combinations of mechanical strength, fracture resistance, biocompatibility, and biological activity, its performance can be further enhanced via a proper modification of material’s surface or by combining the material with some other bioactive ceramics and glass [367]. In addition, as a result of the introduction of Zr into the Ca-Si system, no toxicity was observed. Previous studies confirmed that the optimum content of zirconium and strontium increases the surface energy of the magnesium alloy and enhances the ability to stimulate bone formation around the implant [373,374]. Hydroxyapatite and fluorapatite slurry coated zirconia scaffolds induce osteoconductivity and enhance bonding strength up to 33 MPa [375]. The dispersion of zirconia with alumina lead to produce ZrO_2_-toughened alumina (Al_2_O_3_), known as zirconia-toughened alumina (ZTA) [376]. 

Zirconia (ZrO_2_)/β-tricalcium phosphate (β-TCP) composite has shown excellent mechanical properties and supports osteoblast regeneration [377]. Silk fibroin-chitosan-zirconia (SF/CS/nano ZrO_2_) and chitin–chitosan/nano ZrO_2_ composites provide a suitable environment for cell infiltration and colonization [378,379]. Different temperature based hydroxyapatite-zirconium composites such as 873 K (HZ600), 923 K (HZ650), and 973 K (HZ700) demonstrated that osteoblast growth and mineralization were not influenced by any composite [380]. A new biphasic calcium phosphate (BCP) scaffold reinforced with zirconia (ZrO_2_) was fabricated through the fused deposition modeling (FDM) technique. The 90% BCP and 10% ZrO_2_ scaffold thus created had significantly better mechanical properties than 100% BCP and 0% ZrO_2_ scaffold [381].

**Figure 19:**
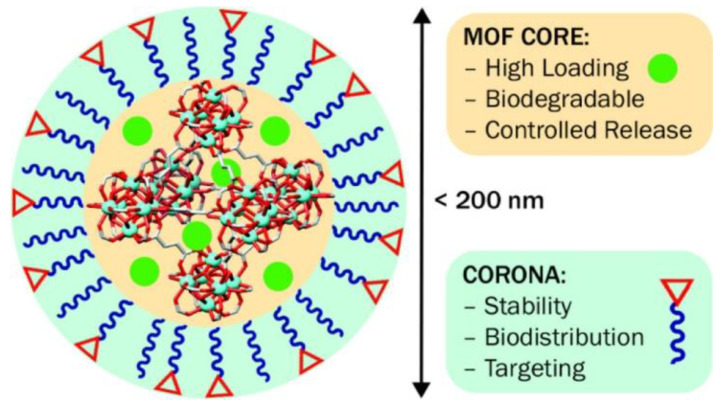
Schematic showing the Zr-fumarate structure with preferred properties of a metal–organic framework (MOF)-based drug delivery device (adapted with permission from © 2018 American Chemical Society [384]).

ZrO_2_ nanoparticle (NP)-doped CTS–PVA–HAP composites (ZrCPH I–III) showed improvement in the tensile strength of ZrCPH I–III with respect to the CTS–PVA–HAP scaffold [382]. Sol–gel cum solvothermal derived mesoporous titanium zirconium (TiZr) oxide nanospheres were used for ibuprofen, dexamethasone, and erythromycin drugs loading and in vitro release studies [383]. The excellent biocompatibility of Zr makes it a good material for metal–organic frameworks (MOFs). Surface functionalization of Zr-fumarate MOF (Figure 19) was used for dichloroacetate (DCA) drug loading, which is more efficient at transporting the drug mimic calcein into HeLa cells [384].

#### 2.2.8. Alumina

Since 1975, the bio-inertness of alumina has been confirmed. Alumina has very high hardness and resistance to abrasion on the Moh scale next to diamond [385]. In addition, the crystalline nature of alumina makes it insoluble at room temperature in regular chemical reagents [386]. Alumina has been used in many fabrications of artificial implants since it was inserted into an artificial femur head in the 1970s [387]. Pure and densified alumina, α-Al_2_O_3_ (corundum), was the first ceramic material used in the biomedical field for dental restorations, cochlear implants, and load-bearing hip prostheses [388]. As porous alumina does not degrade under in vitro and in vivo environments, it may be used for biosensing [389], good electrical insulation [390], and immune isolation [391]. 

Properties such as abrasion resistance, power and chemical inertness favor the use of alumina in hard tissue engineering [392]. If the alumina is implanted in bone marrow, no toxic effects are generated in the surrounding tissue [393]. However, the high stiffness of alumina may lead to have a high elastic incompatibility between the biological tissue and the implant [394]. The tensile strength of alumina can be increased by reducing grain size and increasing its density [395]. In view of their good mechanical behavior, alumina implants are characterized by long-time survival predictions [396]. 

A significant feature in applications involving open and aligned porous structures, such as bone tissue scaffolds, catalysts, and membranes, is the anisotropic nature of porous alumina ceramics [397]. The α-alumina is the most stable oxide amongst transient and metastable types [398]. It should be noted that essential physico-chemical properties of alumina surface are significantly affected by the protein adsorption process. For example, the presence of liquid solutions nearby the implanted site can cause accelerated protein adsorption on the alumina’s surface [399]. Piconi et al. [394] reported the in vitro biocompatibility of alumina with various cell lines such as fibroblasts and osteoblasts, and immunological cells with various cell environments.

The particle size of alumina may affect biocompatibility, particularly when using nanoparticles because of their high surface/volume ratio [400]. Alumina suspensions (70% wt.) and wheat flour (20–30% vol.) were used to synthesize different particle sized porous alumina ceramics [401]. Hydroxyapatite/alumina composite based foam was synthesized via a precipitation method under a variety of pH values that showed a good concentration of Ca^2+^ and PO_4_^3−^ contents [402]. The chemical modification of porous alumina surface with vitronectin and peptide (i.e., arginine-glycine-aspartic acid cysteine (RGDC)) enhanced bone cell adhesion and production of extracellular matrix [403]. 

Porous anodic alumina (PAA) can be fabricated on the surface of other materials through anodization process [404,405]. It can be considered a good nanocontainer to load active agents such as drugs or biomolecules [406]. Evaporation induced self-assembly derived mesoporous aluminum oxide was used for the delivery of poor-water soluble compound Telmisartan (anti-blood pressure drug) with 45% loading efficiency [407]. The drug is not loaded within the pores of the PAA completely, but the surface itself can hold some of this load, which can be quite high; this promotes another phase release [408,409]. 

Calcium phosphate with 20% alumina (Ca_3_(PO_4_)_2_–Al_2_O_3_) bio-ceramic composite revealed enhanced biocompatibility and mechanical properties [410]. Using alumina nanowires reinforcement in polyhydroxy butyrate-chitosan (PHB-CTS/3% Al_2_O_3_) scaffolds enhanced the mechanical properties of the scaffold. The addition of alumina increased by ten times the tensile strength of PHB-CTS/3% Al_2_O_3_, which became higher than its counterpart for the original PHB-CTS scaffold [411]. 

Al_2_O_3_ coating was used for improving the performance of stainless steel 316L and Ti-6Al-4V implants [412]. In general, coating materials are used to protect the surface of the implant material and the interface with the biological system at hand [413]. Nanorod-like HA-coated porous Al_2_O_3_ was fabricated by anodic oxidation that revealed excellent biological activity in vitro [414].

#### 2.2.9. Copper

Copper ions stimulate the proliferation of human vein endothelial cells and mesenchymal stem cells (MSCs) but not human dermal fibroblasts [415,416]. Copper nanoparticles can also act as antifungal and antibacterial agents [417]. Copper is commonly used in bone implants for its antimicrobial activity against a wide range of pathogens [418]. As copper is an essential component of the body, it may be more suitable for in vivo applications [25].

The importance of copper has been studied extensively because its deficiency can lead to osteoporosis [419]. Cu also stimulates angiogenesis and collagen deposition, which are key elements in wound healing [420]. The use of copper-based biomaterials is cost-effective compared to other vital materials based on gold and silver [421]. 

Copper ions were incorporated into biologically active scaffolds for controlled release to improve vascular strengthening and antimicrobial action for prolonged periods [422]. Copper-doped wollastonite (Cu–Ws) particles (1184 nm) have shown biocompatibility towards mouse mesenchymal stem cells (mMSC) up to 0.05 mg/ml concentration [423]. A freeze-dried chitosan/hydroxyapatite/copper-zinc alloy (CS/nHAp/nCu–Zn) composite-based scaffold showed lower degradation and higher protein adsorption without producing toxicity towards rat osteoprogenitor cells [422]. Collagen-copper-doped bioactive glass (CuBG-CS) scaffolds exhibited enhanced mechanical properties (up to 1.9-fold) and osteogenesis (up to 3.6-fold) than chitosan [424]. 

**Figure 20:**
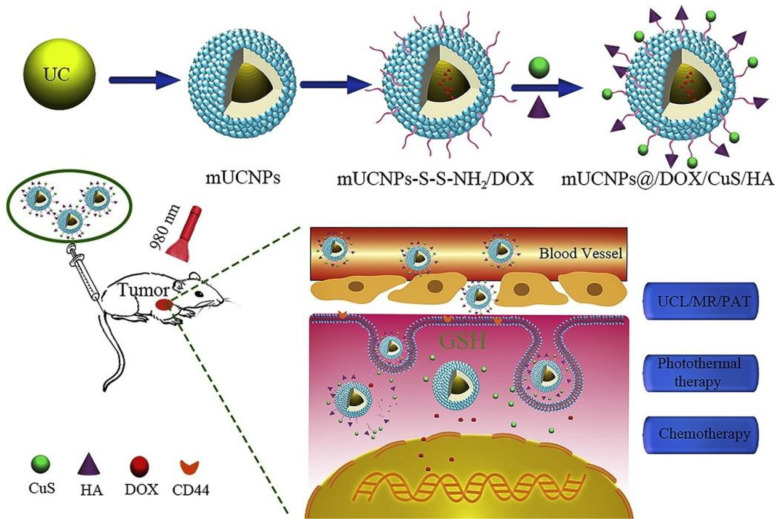
Schematic illustration of the mUCNPs-based redox-stimuli responsive drug delivery system for tumor diagnosis and synergetic chemo-phototherapy (adapted with permission from Elsevier © 2017 [428]).

Copper nanoparticles were investigated also for wound healing applications. 1 μM concentration of 80 nm CuNPs was found not to be toxic to the cultured fibroblast, endothelial, and keratinocyte cells, and it supported endothelial cell migration and proliferation [425]. CuNPs may alter the structure of proteins and enzymes, affecting their normal functions and causing inactivation of bacterial functions at the injury site [426]. Chen et al. reported the cytotoxic effect of copper NPs towards human histolytic lymphoma (U937) and human cervical cancer cells by inducing apoptosis [427]. CuNPs were used to design a special drug delivery system for chemotherapy. For example, Figure 20 illustrates a mesoporous, upconversion, nanoparticles (mUCNPs)-based controlled-release drug carrier system exhibiting higher upconversion luminescence emission intensity [428].

#### 2.2.10. Magnetic Nanoparticles

Magnetic elements (i.e., iron, nickel, cobalt, and their oxides) were utilized for the fabrication of nanomaterials for different medical applications [429,430] such as MRI, drug delivery, medical diagnostics, cancer therapy, biosensoring, and magneto-optic devices. Magnetic nanoparticles can be synthesized through different techniques including co-precipitation [431], microemulsion [432], hydrothermal synthesis [433], sol–gel process [434], polyol synthesis [435], flow injection [436], sonolysis/sonochemical methods [437], microwave irradiation [438], electrochemical synthesis [439], solvothermal method [440], chemical vapor deposition [441], laser pyrolysis [442], and green synthesis [443] using biomass or biotemplate. 

**Figure 21:**
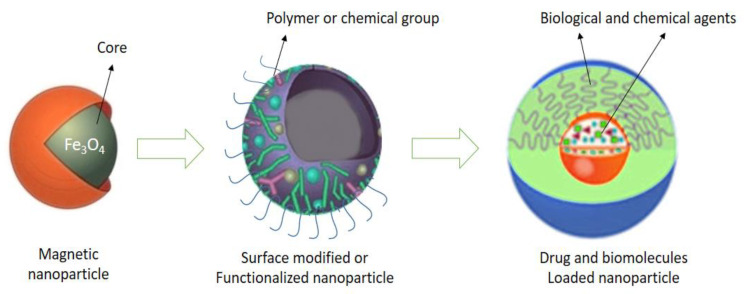
Surface modified magnetic nanoparticle.

Due to high magnetic flux density, magnetic nanoparticles were used for drug targeting [444] and bio-separation [445], including cell sorting [446]. Sun et al. [447] analyzed metallic, bi-metallic, magnetic cationic liposomes and superparamagnetic iron oxide nanoparticles for imaging and drug delivery. The surface of magnetic nanoparticles also needs to be functionalized to recognize specific targets (Figure 21) [448]. Polyethylene glycol (PEG) is one of the best polymers used for the functionalization of magnetic nanoparticles by surface modification [449]. Interestingly, surface modified magnetic nanoparticles reduce nonspecific interaction with biological molecules.

Magnetic manipulation is another important advantage of magnetic nanoparticles [450]. It is done by labeling cells with magnetic nanoparticles that can easily be controlled by remote control or external magnetic field [451]. The magnetic nanoparticles, which are usually smaller than 10 nm can be easily transported through skin lipid matrix and hair follicles to the stratum granulosum, where it is condensing between corneocytes [452]. 

In orthopedic surgery, implant-associated infection is a serious issue, as stated in the previous sections. Infection around a bone graft can lead to serious illness or failure of surgery. Drug-loaded Fe_3_O_4_ composites promote cell adhesion, proliferation, and osteogenic differentiation of hBMSCs [453,454,455]. In stem cell therapy for bone regeneration, an application of these NPs is the magnetic targeting of stem cells to the deserved locations, known as magnetic homing of stem cells. For example, penetration of ferumoxide-labeled cells into porous hydroxyapatite ceramic implanted in a rabbit ulnar defect was significantly facilitated by this approach, which improved bone formation even in the chronic process [456].

#### 2.2.11. Summary and Statistical Analysis of the Survey on Inorganic Nanobiomaterials 

The survey on inorganic nanobiomaterials presented in Section 2.2 covered some 230 articles, practically the same as its counterpart for organic biomaterials not counting about 30 articles on fabrication techniques of silica and magnetic nanoparticles.

While the number of technical papers appears to be rather uniformly distributed among the ten types of inorganic nanomaterials considered in this survey, it should be noted that most studies focused on nanoparticles and their functionalization for drug/gene/therapy delivery, cell labeling, biosensing, and bioimaging (75%), followed by studies on development and fabrication of new composite materials and scaffolds (25%).

Gold and titania present the largest variety of nanostructures and the latter material may also be available in the form of nanotubes. Gold nanoparticles may represent the best solution for most applications in view of the possibility of controlling size and dimensions of nanostructures as well as for their special physical properties (for example, local plasmon resonance). However, massive utilization of GNPs is obviously limited by the high cost of gold. Silica and titania nanoparticles also are widely utilized as standalone materials or in combination with gold and silver nanoparticles.

Similar to what has been observed for organic nanobiomaterials, a rapidly growing research area in the field of inorganic nanobiomaterials for bone tissue engineering is to hybridize them with other materials (e.g., chitosan, PLA, PLGA, collagen, and hydroxyapatite) to enhance mechanical properties, biocompatibility and osteogenetic properties of the modified materials. Development of high-performance scaffolds comprised of multiple materials is the final stage of this complicated process.

## 3. Applications of Nanobiomaterials

**Figure 22:**
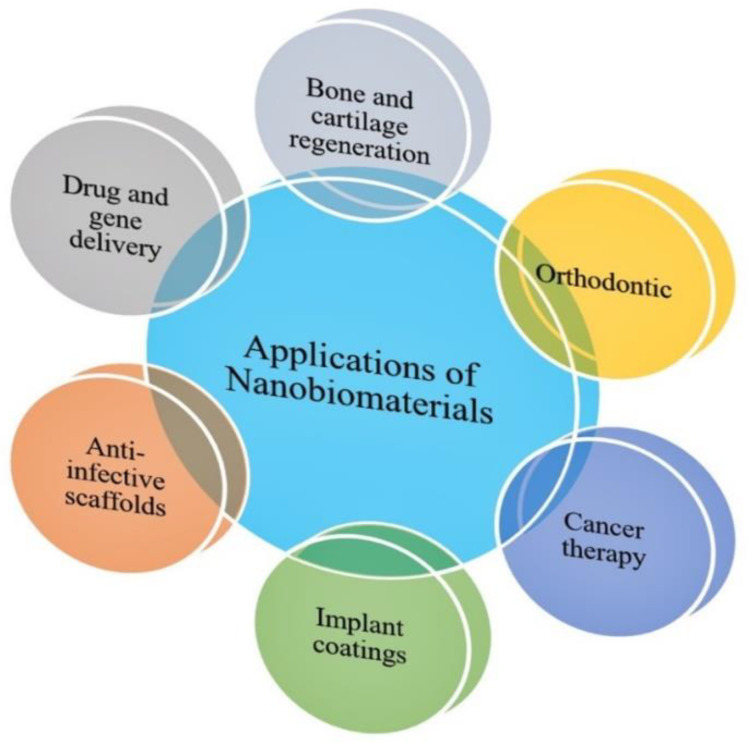
Applications of nanobiomaterials in the biomedical field.

Nanobiomaterials have outstanding mechanical, chemical, electrical and optical properties, which make them highly suited for a variety of biological applications [70]. Nanotechnologies made it possible to develop new nanoscale materials (nanobiomaterials) with upgraded surface area to volume ratio, enabling more surface interactions [457,458,459]. As nanobiomaterials possess very specific properties that may be tailored to specific targets (i.e., solubility (for otherwise insoluble drugs), carriers for hydrophobic entities, multifunctional capability, active and passive targeting, ligands (size exclusion), and reduced toxicity), they have tremendous potential for disease identification (as imaging tools), care delivery, and prevention in new ways [107]. Nanobiomaterials are special kinds of materials that are introduced into the body for the treatment of damaged hard tissues [460]. The huge variety of biomedical applications of nanobiomaterials are illustrated in Figure 22 [428,461,462,463].

Nanobiomaterials have well-defined nanostructures such as size, shape, channels, pore structure, and surface domain [464]. Nanoscale dimension enables nanobiomaterials to develop critical physical and chemical characteristics that enhance their performance [465,466]. The properties and behaviors of nanobiomaterials, therefore, allow the diagnosis, monitoring, treatment, and prevention of diseases [467]. Nano-size materials show more catalytic reactions at their surface than macro-sized or conventional materials [468]. The nanoscale biomaterials create biomimetic feature towards most of the proteins which support further biological reactions such as cell attachment, growth, proliferation and generation of new tissue [36]. 

### 3.1. Bone Regeneration

**Figure 23:**
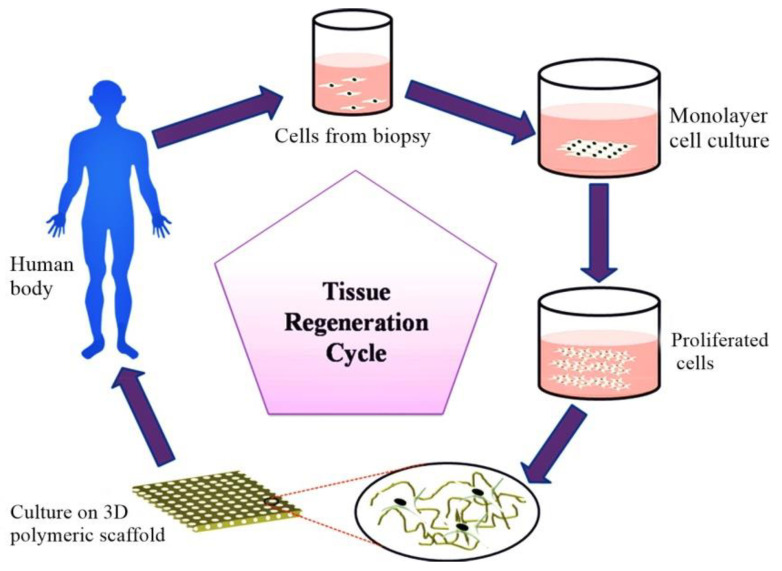
Basic principle procedures for tissue engineering. (Adapted with permission from ©2019 American Chemical Society [255]).

A perfect bone and cartilage repair scaffold materials should neither suppress the activity of normal cells nor induce toxicity during and after implantation [469]. Figure 23 illustrates the basic cycle of tissue regeneration using nanobiomaterials or derived scaffolds [255]. 

The various synthetic nanostructured matrices are able to stimulate cell differentiation with a focus on preserving the structural features, composition, and biology of natural bone tissue [470]. The main constituents used so far in this regard are nano-hydroxyapatite [471], anodized titanium [472], collagen [473], and silver-incorporated calcium silicate. Nanobiomaterials (1–100 nm) generated from polymers, metals, ceramics, and composites act as effective constituents for hard tissue and play a significant role in osteointegration on nanostructured surfaces [474]. 

Alumina has been widely used for the fabrication of knee and hip joint prosthesis with low wear rates [475]. Bioactive glasses were used as a prosthesis for the restoration of the ossicular chain of the middle ear and oral implant to preserve the alveolar ridge from bone resorption [476,477]. Different metal oxides such as ZnO, Fe_2_O_3_, TiO_2_, and Al_2_O_3_, and polymers such as PLA, PGA, and their copolymers were used with bioactive glass systems for hard tissue engineering applications [478,479].

### 3.2. Drug Delivery

**Figure 24:**
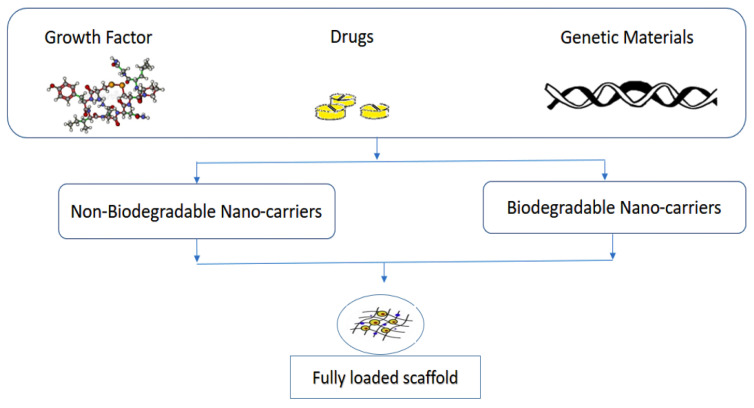
Drugs and biomolecules loaded scaffold for tissue engineering.

As mentioned above, various nanobiomaterials can be used for bone regeneration, prevention of infections, and osteointegration [480,481]. A nanoparticle that functions as carrier can stabilize the bioactive molecules through encapsulation [482], facilitating targeting cellular delivery and targeted drug release [483,484]. Nanospheres, tubes and capsules are widely accepted tools for targeted and sustained release drug delivery because of their small size and high specific surface area, which encapsulates the drug molecules and shows high reactivity to the surrounding tissues [485]. The materials selected for nanosphere fabrication depend on application principles and requirements. Some factors in this regard include size, drug characteristics, surface properties, biodegradability and biocompatibility of materials and drug release profile [486]. The 2D and 3D structures of scaffolds can be useful for the drug (poorly soluble drugs) loading purpose in tissue engineering (Figure 24). 

### 3.3. Gene Delivery

**Figure 25:**
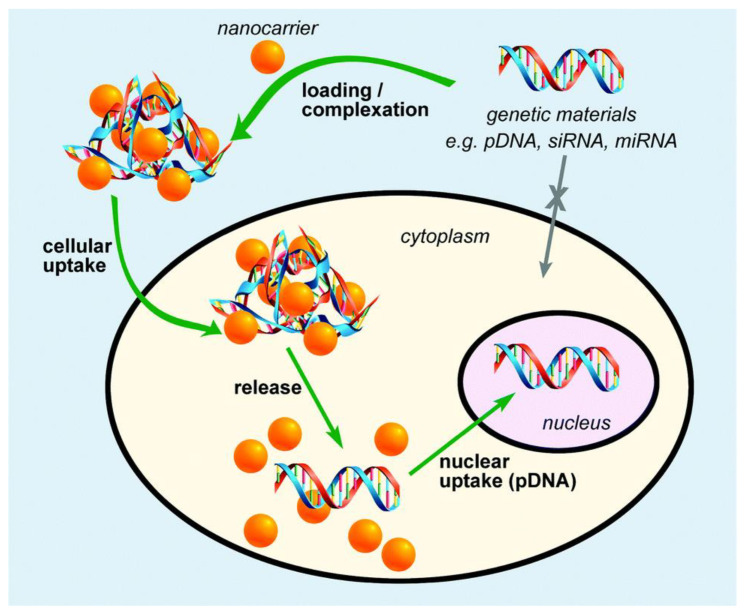
Fundamental steps of gene delivery by nanocarriers (orange spheres). (Adapted with permission from ©2016 Royal Society of Chemistry [489]).

The rapid development of nanotechnology made available novel DNA and RNA delivery systems for gene therapy (Figure 25) that can be used instead of viral vectors [487]. Gene therapy collectively refers to therapies aimed at manipulating gene expression in living organisms by supplying exogenous DNA or RNA that is incorporated or not incorporated to cure or prevent diseases [488]. There is great incentive to work towards safer and targeted viral vectors and to engineer more effective non-viral systems that can achieve secure, effective gene therapy in humans because of the enormous potential for gene therapies to influence medicine.

There are a number of nanocarriers used for gene delivery (Figure 25) [489] applications which are based on lipids [490,491], liposomes [492,493,494], dendrimers [495], polymers [496,497], graphene [498,499], carbon nanotubes (CNTs) [500,501], mesoporous silica [502], gold nanoparticles [503,504], magnetic nanoparticles [505,506], and other types of inorganic nanoparticles. 

The number of clinical trials for cancer [507], liver disease [508], hemophilia [509], and bone regeneration [510,511,512] is continuously increasing due to the promising opportunity to correct gene disorders. Nanomaterials are used for gene delivery because of their small size and superior stability [513]. Before the use of these nanomaterials, surfaces need to be functionalized with small biomolecules or polymers to adapt their physiochemical properties such as hydrophobicity, charge density, and binding affinity [514,515]. Factors including molecular weight, biodegradability, rigidity, charge density, pKa value, solubility, crystallinity, and hydrophobicity ensure effective and safe gene delivery [516,517]. 

**Figure 26:**
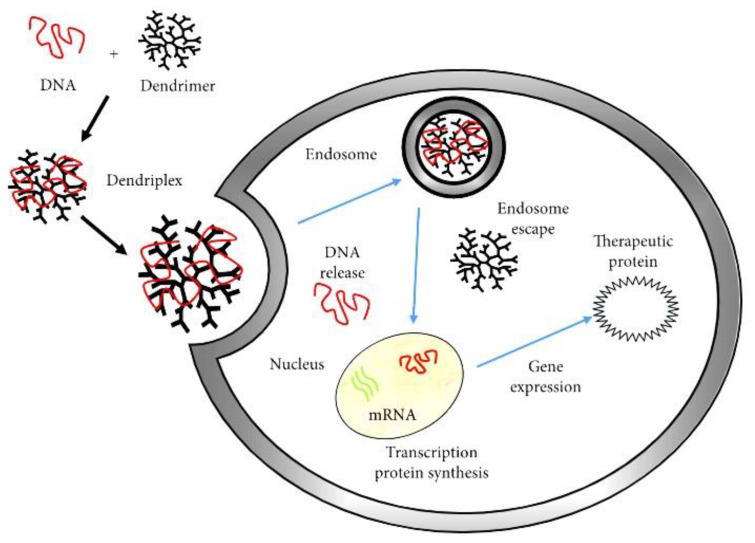
Schematic diagram for a possible route in the use of dendrimers as gene delivery vectors.

Surface-modified graphene oxide through cationic polymers such as polyethylenimine (PEI) provides a large surface area for the encapsulation of DNA molecules [518]. DNA/drug molecules attached graphene oxide conjugated provide an outstanding platform for the immobilization of nucleotides on its surface [519]. Mesoporous silica nanospheres (MSNs) and functionalized single-walled carbon nanotubes (SWCNT) represent an excellent gene delivery system [520]. In Figure 26, a potential route is recorded for the use of dendrimers as vectors of gene delivery. As plasmid DNA penetrates the cell membrane, it makes (in vitro) a complex between dendrimer and DNA (called dendriplex). This complex is transported through the blood system to the specific cell. Finally, the DNA moves through the cytoplasm to reach the nucleus for gene expression in series [521,522].

### 3.4. Anti-Infective Nanobiomaterials

Disease, injury, and trauma can lead to serious bacterial infections, which cause disease and adverse complications in host tissues and even death of patients [523]. Nanobiomaterials made from polymers, metals, and ceramics might be a potential source of infection when they are introduced into the body [524,525]. Virus and bacterial infections cause unregulated damage that leads to organ failure [526]. In polymeric biomaterials, the most common bacterial infections are powered by *Staphylococcus epidermidis (S. epidermidis)* from skin and *Staphylococcus aureus (S. aureus)*, which may identified on metallic biomaterials [527]. Ceramics and metals can represent an alternative because of their resistance to infection. However, in presence of minor imperfections on the surface or microfractures, pathogens, such as bacteria, can form a colony [528]. Biomaterials from natural sources were used as alternative as scaffolds for promoting regeneration but they carry a risk for pathogenic transmission [529]. 

### 3.5. Nanobiomaterials for Coating 

Micro/nanoscale tissue engineering scaffolds play a vital role on the organization of natural extracellular matrix [530]. Nanostructured 3D scaffolds enhance cell functioning, migration, differentiation, proliferation, and extracellular matrix formation [531]. 

Nanobiomaterials used for coatings include silica (SiO_2_), titania (TiO_2_), zirconia (ZrO_2_), alumina (Al_2_O_3_), zinc oxide (ZnO), CNT, graphene, and various combined oxides [532]. Simple calcium phosphate coating method on metals, glasses, inorganic ceramics and organic polymers (such as PLGA, PS, PP, and silicone), collagens, and silk fibers can improve biocompatibility or enhance the bioreactivity for orthopedic applications [494,533]. TiO_2_ and Al_2_O_3_ can be used as biologically active coating agents, supporting cell adhesion, growth, osteogenic differentiation, bone matrix production, and mineralization [534]. Nanostructured TiO_2_ has a positive effect on the performance of bone cells. TiO_2_ is available in the form of nanocrystals [535], nanofibers [536], nanoparticles [537], also immobilized on nanotubes [538]. TiO_2_ nanotube coating on any substrate enhances hydroxyapatite formation in SBF [539]. Nano silica coating on Ti-6Al-4V alloys generates apatite and supports adhesion and attachment of human osteoblast-like Saos-2 cells [540]. Nitinol coated stainless steel has shown enhanced biocompatibility but Ni ions produce an allergic response and toxicity [541]. Zirconia coated pure and yttrium-stabilized nanostructure promote deposition of apatite from SBF, which supports cell adhesion and growth [542]. Zinc oxide doped with alumina or functionalized with the silane coupling agent KH550 supports the proliferation of fibroblasts [543].

Carbon nanotubes have been used with various synthetic and natural polymers or minerals for the improvement of mechanical properties [544]. CNT and other nano-carbon forms stimulate cell adhesion and growth of osteogenic cells. Graphene-based films and composites used for biomaterial coatings can be obtained from pure or oxidized graphene. These graphene-based films improve the osteogenic differentiation manifested by collagen I and osteocalcin, high calcium phosphate deposition, and high alkaline phosphatase activity [545,546]. Due to the antimicrobial impression of graphene, graphene oxide (GO), and their derivatives, these materials can be used for implant coating [547]. Graphene oxide (GO) coating on the collagen scaffold induces morphological changes depending on GO concentration [548]. The application of GO improved physical properties like compressive strength as well as adsorption of Ca and proteins without changing porosity [549]. Graphene oxide-silk fibroin (GO-SF) composite used as an alternative to coating with collagen, showed improved biomechanical properties and proved could work in cellular environments [550]. 

HA can accelerate new bone formation by coating on titanium and tantalum scaffolds. It was demonstrated that after 6 weeks of implantation with titanium and tantalum scaffolds coated is possible to reach fully dense bone formation [551]. Calcium-phosphate-coated Fe foam showed better differentiation and proliferation rate of human mesenchymal stem cells than uncoated Fe foam [552]. Polymer-coated mesoporous silica nanoparticles are effective, cell-specific targeted chemotherapeutic agent delivery method [553]. In rat calvarial defects, HA-coated PLGA scaffolds alone promote bone regeneration and increased exposure to HA nanoparticles on the scaffold surface has been documented to result in accelerated bone deposition by local progenitors [554].

### 3.6. Nanostructured Scaffolds

**Figure 27:**
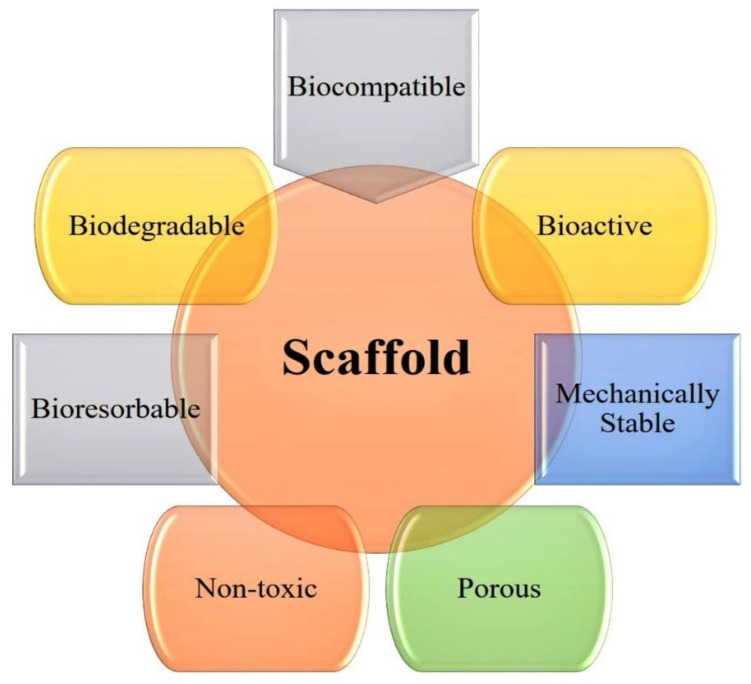
Desired properties of an ideal scaffold.

Scaffolds are artificial constructs that provide support, tensile strength, and aid in tissue ingrowth [555]. They can also serve as carriers for growth factors, drugs and other required ingredients [556]. Scaffolds mimic the presence of extracellular matrix and allow the replacement of tissue without producing any harmful disturbance with respect to surrounding tissues. An ideal scaffold should be biocompatible, biodegradable, bioactive, non-toxic, mechanically stable, biodegradable, and bioresorbable (Figure 27) [557]. The amalgamation of organic and inorganic materials with scaffolds may enhance morphology and mechanical properties, thus supporting better cell attachment and proliferation [558].

Scaffold properties can be improved by using nanoparticles because organic and inorganic minerals in natural bone have nanoscale structures [559]. Many studies found that the addition of titanium and iron improve biological and mechanical properties such as collagen synthesis and apatite generation [560,561]. In addition, engineered nanofibrous scaffolds are also suitable for loadbearing applications and can replace natural extracellular matrix (ECM) with artificial ECM. The nanofibrous scaffold can therefore get a much more suitable environment for cellular growth and eventual regeneration of the bone [562]. Nanofiber-based scaffolds have been fabricated by using different synthetic polymers including PCL [563,564,565,566], PLLA [567,568], copolymer [569], PLGA [61], and chitosan [569]. 

Different kinds of metallic nanoparticles can be used for the synthesis of composite-based scaffolds with enhanced mechanical characteristics, cell adhesion, and bone tissue generating capacity [12]. The incorporation of titanium, iron, and alumoxane in a scaffold can improve mechanical properties, collagen synthesis, calcium deposition, and alkaline phosphatase activity [561]. 

Graphene and its derivatives were used as reinforcement material for fibrous scaffolds, films, and hydrogels [570]. The graphene and graphene oxide incorporation into hydrogels yield enhancements in mechanical properties without producing adverse effects on encapsulated fibroblast cells [571]. Carbon-based nanomaterials can be used to improve mechanical strength of scaffolds [572]. Alumina, titania, bioglass, and hydroxyapatite support osteoblast adhesion and growth [573]. 

**Figure 28:**
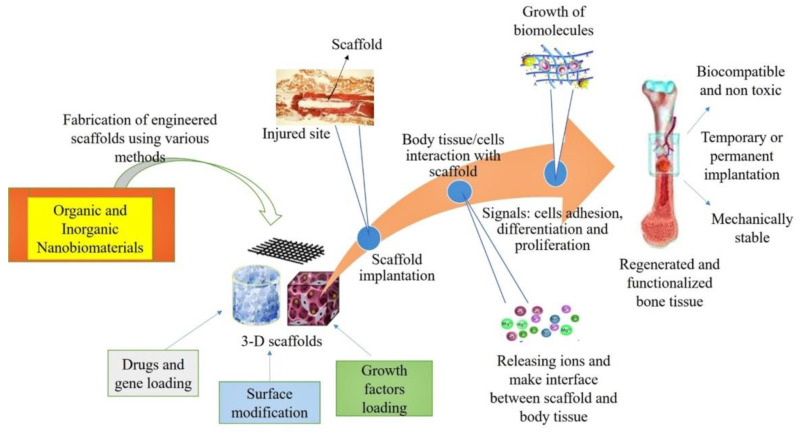
Engineered organic and inorganic nanobiomaterials for hard tissue engineering applications.

Nanobiomaterial-based composite structures are an efficient platform for the synthesis of engineered scaffolds and application in bone tissue engineering (Figure 28) [574]. Nanocomposite-based scaffolds exhibit inherent characteristics such as porous and rough surface and increased wettability, which promote fast bone regeneration. These nanocomposite-based scaffolds provide a porous structure for nutrients exchange and increased protein adsorption. Scaffolds exhibited micro/nano-scaled porous structural pathway for cell–scaffold interaction and integrin-triggered signaling pathway. The nanoscale features support bone cells (osteoblast) and bone-derived stem cells proliferation, migration, cell signaling, stem cell fate, and genetic cell fate. The nanobiomaterials based scaffold have notable mechanical and biological advantages and can induce bone tissue regeneration [531]. The nanostructured materials improve morphological characteristics of scaffolds that may enhance osteoinduction, bone cell attachment, differentiation, proliferation, and natural bone cell growth within the extracellular matrix [12]. 

Hydroxyapatite (HA) has attracted attention because of its inherent biological compatibility and bone conduction as well as its similarity with bone minerals [575]. For this reason, HA was combined with a number of synthetic and natural polymers such as polycaprolactone [576], poly (lactic acid) (PLA) [577], polyethylene, poly(lactic-co-glycolic acid) (PLGA) [203], collagen [578], gelatin [148], and chitosan [579] to fabricate scaffolds. These composite based scaffolds showed improved mechanical properties, porosity and biocompatibility without or with significantly less adverse effects.

### 3.7. Bone Cancer Therapy

**Figure 29:**
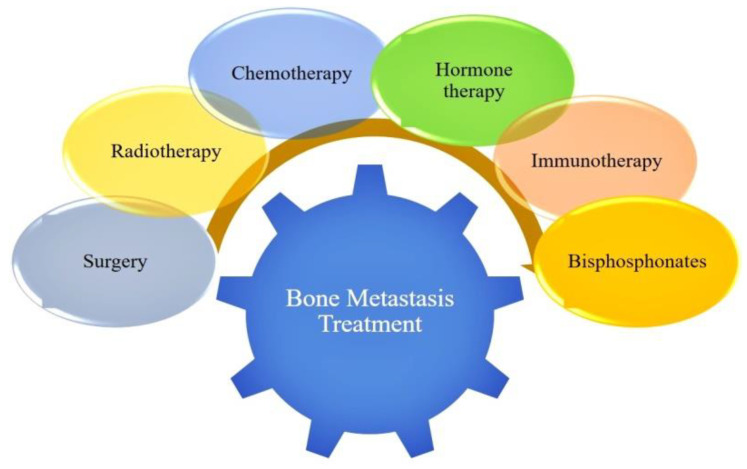
Bone metastasis management through combination of therapies.

Cancer is the uncontrolled growth of tissues that could lead to invasion into other organs without proper regulation or differentiation [580]. Conventional cancer therapy is associated with multiple adverse side effects [581]. Bone metastases or “bone mets” occur when cancer cells from the primary tumor relocate to the bone and also spread in the prostate, breast, and lung, which leads to painful (75% of patients) and devastating skeletal-related events (SREs) [582,583]. Depending on the stage of the disease, history, and the overall health of the patient, disease management includes a combination of therapies as shown in Figure 29 [584].

**Figure 30:**
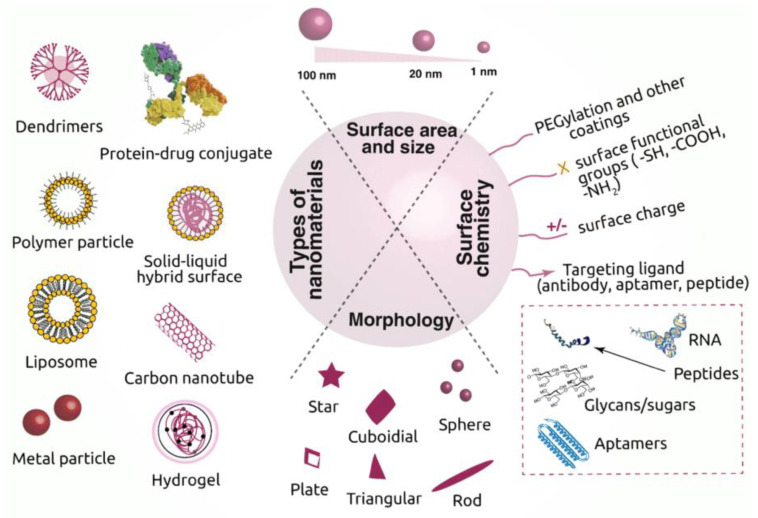
Schematic representation of different types of nanomaterials and their drug-loaded conjugates employed in cancer therapy. (Adapted with permission from ©2019 Springer [589]).

The different types of nanoparticles (NPs) used as carriers for small-molecule drugs, proteins, and nucleic acids [585] can be localized to specific disease locations for the treatment of bone metastasis [586]. Nanoparticles also improve the efficiency of other methods used for treating bone metastasis [587]. The effectiveness of NPs depends on their accumulation in vascularized solid tumors via the enhanced permeability and retention (EPR) effect [588]. A wide variety of nanomaterials have been developed in the 1 to 100 nm range and include various anti-tumor drugs (Figure 30) by fine-tuning the chemical structure, scale, and shape (morphology) that can regulate the nanomaterials’ functionality [589].

## 4. Counter-Indications

There is inevitably some sort of interaction between the organic or inorganic materials and the biological environment when individual or composite biomaterials are put in contact with the tissues and fluids of the human body. The basic clinical research may decide that the materials should not cause any local or systemic adverse reactions. Recent studies exposed that nanosized materials can easily penetrate biological membranes of normal cells and enter vascular system to facilitate redistribution in different tissues. Nanomaterials, which by themselves are not very harmful, could become toxic if are ingested in higher concentration. The toxicity of metal based biomaterials to the liver is an important basis for the safety assessment of nanosized materials. Metal based nanoparticles release ions which may enter the cells and affect the functions of organelles, leading to liver injury. Various factors including amount, composition, pH, and fabrication techniques may decide the compatibility and cytotoxicity of biomaterials. The research is ongoing to improve the existing technologies which may produce highly compatible substitutes without producing adverse effects.

## 5. Conclusions

This review article provided a broad overview of the various types of organic and inorganic nanobiomaterials and their applications in the field of hard tissue engineering. Besides classifying nanobiomaterials, the survey covered several key aspects like bone/cartilage regeneration, drug/gene delivery, anti-infection properties, coatings, scaffold fabrication, and cancer therapy. A total of 550 articles selected by means of web search engines widely used in science and engineering were reviewed in this study. Interestingly, the number of reviewed articles was approximately the same for organic and inorganic biomaterials.

Biomaterials science is a highly multidisciplinary area. Developments in life science and nanotechnology enabled scientists and engineers to conceive new designs and improve the existing bone structure. For example, advances in nanotechnology allowed for the development of novel methods for fabricating new nanostructured scaffolds possessing a higher efficiency in tissue regeneration.

Nanomaterials represent an excellent tool for research and therapeutic approaches in bone tissue engineering. Organic nanomaterials are more biocompatible, nontoxic, and help more with cell regeneration than inorganic nanomaterials. However, inorganic nanomaterials provide better mechanical strength and inertness to chemical agent. From the references cited in this survey it appears that nanoparticles, graphene and nanocomposites are the most diffused types of nanostructures used for hard tissue applications. An important research trend which results in a rapidly growing number of published articles is the development of new composite nanobiomaterials especially for scaffold applications.

Interactions between bone cells and nanomaterials depend on the composition of nanoparticles. Proper selection of nanoparticles may result in faster bone regeneration and recovery. Besides composition, the overall performance of a nanobiomaterial depends on porosity, microstructure, mechanical properties and functionality. Nanomaterials-based scaffolds also play a major role in three-dimensional tissue growth. Nanostructural modifications provide a favorable environment for bone regeneration. 

The survey presented in the article proved that tissue engineering supports (i) application of engineering design methods to functionally engineered tissues, (ii) development of novel biomaterials for constructing scaffolds that mimic extracellular matrix, and (iii) creating artificial microenvironments. Nanobiomaterials represent an excellent tool for research and therapeutic approaches in bone tissue engineering. However, further investigations should be aimed at producing advanced nanobiomaterials suitable for hard tissue engineering that can fill the gap between biomaterial fabrication and clinical implementation.

## Figures and Tables

**Table 1:** Types of organic nanobiomaterials with their applications.

Types of Nanomaterials	Size (nm)	Applications	References
Lipid	<100	Nanocarriers for anticancer drug doxorubicin Osteoblastic bone formation Osteoporosis treatment	[42,43,44,45]
Liposome	>25	High encapsulation of hydrophilic drug (drug delivery) Growth factor delivery Therapeutic gene delivery Used as a template	[46,47,48]
Dendrimers	<10	Multidrug delivery system	[46,49,50]
Chitosan	20–200	Nano/microparticles or fiber-based scaffolds Drug delivery Support chondrocyte adhesion Implant coating	[51,52,53,54,55]
Collagen	–	Drug Delivery Scaffolds	[56]
Gelatin	<200	Bone scaffold systems formation Drug-loaded gelatin nanoparticles (DGNPs) Promote cell growth	[24,57,58]
Poly(lactic-co-glycolic acid) PLGA	100–250	Drug delivery Scaffold system Nanostructured Film Enhanced cell attachment and growth	[59,60,61,62]
Carbon Nanotubes	20–100	Drug delivery Biosensing Mechanically improved scaffold fabrication Enhanced rat brain neuron response	[63,64,65,66]

**Table 2:** Types of inorganic nanomaterials with their applications.

Types of Nanomaterials	Size (nm)	Applications	References
Nano Silica	10–100	Composite-based scaffold Bio-imaging Drug delivery Enhanced osteogenic differentiation	[225,226,227]
Gold nanostructured materials	5–50	Bioinorganic hybrid nanostructures Thin film scaffold Bio-imaging	[228,229,230]
Magnetic nanomaterials and nanoparticles	10	Drug and gene delivery Improved cell adhesion Cell tracking	[21,231,232]
Bioactive Glasses	20–500	Improved scaffolds performance Drug and gene delivery	[233,234]
Silver nanoparticles	1–100	Tissue repair and regeneration Antibacterial action	[235,236,237]
Nanostructured Titanium	<300	Nano tubular anodized titanium Improved mechanical properties Enhanced chondrocyte adhesion Support osteoblast adhesion and proliferation Orthopedic coating	[238,239,240,241,242,243]
Hydroxyapatite	20–80 ~200–500	Enhanced osteoblast functioning Increase bone apatite formation	[244,245]
Zirconia nanoparticles	<100	Enhanced osteointegration Antibacterial implants formation	[246,247]
Alumina nanoparticles	<80	Enhanced bone cells adhesion and proliferation Calcium phase deposition	[245,248]
Copper nanoparticles	<100	Antimicrobial implant fabrication	[25]

**Table 3:** Mineral composition of hydroxyapatite, bone, and teeth.

Types	Ca	P	Ca/P	Total Inorganic (%)	Total Organic (%)	Water (%)
**HA**	39.6	18.5	1.67	100	-	-
**Dentine**	35.1	16.9	1.61	70	20	10
**Bone**	34.8	15.2	1.71	65	25	10
**Enamel**	36.5	17.1	1.63	97	1.5	1.5
